# Oxygenase-Catalyzed Desymmetrization of *N*,*N*-Dialkyl-piperidine-4-carboxylic Acids**

**DOI:** 10.1002/anie.201406125

**Published:** 2014-08-27

**Authors:** Anna M Rydzik, Ivanhoe K H Leung, Grazyna T Kochan, Michael A McDonough, Timothy D W Claridge, Christopher J Schofield

**Affiliations:** Department of Chemistry, University of Oxford, Chemistry Research Laboratory12 Mansfield Road, Oxford OX1 3TA (UK); Structural Genomics Consortium, University of Oxford, Old Road Campus, Roosvelt DriveHeadington OX3 7DQ, United Kingdom

**Keywords:** biocatalysis, γ-butyrobetaine hydroxylase, C–H activation, desymmetrisation, oxygenases

## Abstract

γ-Butyrobetaine hydroxylase (BBOX) is a 2-oxoglutarate dependent oxygenase that catalyzes the final hydroxylation step in the biosynthesis of carnitine. BBOX was shown to catalyze the oxidative desymmetrization of achiral *N*,*N*-dialkyl piperidine-4-carboxylates to give products with two or three stereogenic centers.

Enzyme catalyzed desymmetrization of organic compounds is of considerable synthetic utility.[1] Enzymes that catalyze redox reactions have been used as biocatalysts,[2] including for desymmetrization;[1], [3] however, there are few examples of the use of isolated enzymes involved in the oxidation of unactivated C=H bonds for this purpose.[4] Given the breadth of oxidative reactions catalyzed by non-heme oxygenases, these enzymes hold considerable potential for biocatalysis, including for late-stage oxidations. Recently, we reported that the ferrous iron and 2-oxoglutarate (2OG) dependent oxygenase γ-butyrobetaine hydroxylase (BBOX) can accept substrates other than γ-butyrobetaine (GBB),[5] and in some cases catalyzes unusual oxidative rearrangements.[5], [6] BBOX catalyzes the final hydroxylation step, the C3 hydroxylation of GBB, in the biosynthesis of carnitine, which is required for the transport of fatty acids into mitochondria[7] (Scheme 1 A). We report that BBOX can catalyze the oxidative desymmetrization of achiral *N*,*N*-dialkyl piperidine carboxylic acids to give products with two or three stereogenic centers.

**Scheme 1:**
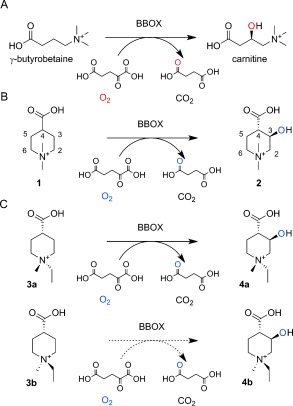
BBOX catalyzes oxidative desymmetrization. A) BBOX catalyzes the stereoselective hydroxylation of γ-butyrobetaine (GBB) to (3*R*)-carnitine during carnitine biosynthesis. B) The BBOX-catalyzed oxidation of achiral *N*,*N*-dimethyl piperidine-4-carboxylic acid (1) gives (3*R*)-hydroxy-(4*S*)-*N*,*N*-dimethyl-piperidine-4-carboxylic acid (2). In the BBOX-catalyzed oxidation of a mixture of *cis*-*N*,*N*-ethylmethyl-piperidine-4-carboxylic acid (3 a) and *trans*-*N*,*N*-ethylmethyl-piperidine-4-carboxylic acid (3 b), only the *cis* isomer undergoes effective hydroxylation to give (3*R*)-hydroxy-(*1R*)-*N*,*N*-dimethyl-piperidine-(4*S*)-carboxylic acid (4 a).

To explore the biocatalytic potential of BBOX, a set of cyclic GBB analogues was screened as potential substrates (Figure S1 in the Supporting Information) by using a mass spectrometry (MS)-based assay. The screen led to the identification of *N*,*N*-dimethyl-piperidine-4-carboxylic acid acid (**1**) as a potential human BBOX substrate (Figure S2). NMR analysis of the reaction product resulting from the incubation of *N*,*N*-dimethyl-piperidine-4-carboxylic acid (**1**) with BBOX revealed the formation of a single alcohol product: 3-hydroxy-*N*,*N*-dimethyl-piperidine-4-carboxylic acid (**2**; Scheme 1 B, Figure 1, and Figure S3). The product stereochemistry was assigned as (3*R*)-hydroxy-(4*S*)-*N*,*N*-dimethyl-piperidine-4-carboxylic acid based on coupling constant analysis, which revealed that the C3=H and C4=H bonds are both axial (H4: *J*_aa_=12.4 Hz, *J*_aa_=10.5 Hz, *J*_ae_=4.7 Hz; H3: *J*_aa_=10.8 Hz×2, *J*_ae_=4.7 Hz), and protein crystallographic analysis (see below). Kinetic analysis revealed a *K*_M_ value of 40 μm and a *k*_cat_ value of 0.14 μm s^−1^ when using **1** as the BBOX substrate, compared to values of 4 μm and 0.83 μm s^−1^ for GBB (Table S1). Interestingly, substrate inhibition was observed only above 0.2 mm with **1**, whereas substrate inhibition occurs at >20 μm of GBB[8] (Figure S14).

**Figure 1:**
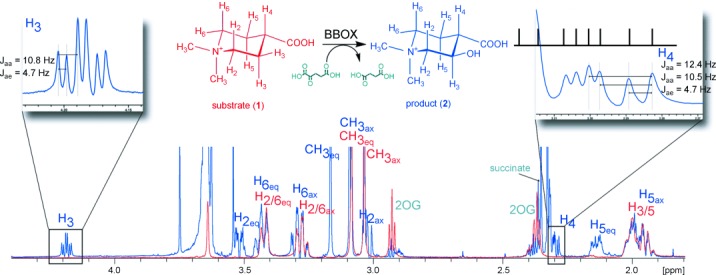
^1^H NMR assignments of the BBOX-catalyzed hydroxylation of achiral *N*,*N*-dimethyl piperidine-4-carboxylic acid (1). An overlay of the ^1^H NMR spectrum of a reaction mixture containing 1 prior to (red) and after (blue) BBOX incubation reveals the formation of a new species (2). The pattern of the coupling constants for H3 and H4 of 2 demonstrate that the C3=H and C4=H bonds are both axial. The signal for C4=H partly overlaps with other signals of the reaction mixture.

To investigate the binding mode of **1**, we obtained a crystal structure of BBOX in complex with **1**, *N*-oxalylglycine (NOG, a structural mimic of 2-oxoglutarate), and Ni^II^ (an Fe^II^ surrogate) at 1.9 Å resolution (H32 space group; PDB ID: 4CWD). The overall fold of BBOX in the new structure is very similar to that obtained for the human BBOX structure in complex with GBB, NOG, and Zn^II^ ions[5] (PDB ID: 3O2G; Figure S4), with the mobile active-site loop in the “closed” conformation (as is proposed to be required for efficient substrate turnover [8a]). The active-site residues in the BBOX **1** NOG Ni^II^ complex are positioned similarly to those in the BBOX GBB NOG Zn^II^ complex (Figure 2). At the active site, the piperidine ring of *N*,*N*-dimethyl-piperidine-4-carboxylic acid (**1**) is bound in a chair conformation, with its carboxylate group in the equatorial position (Figure 2). This result is consistent with the preferred conformation of **1** as determined by solution-phase NMR analysis (Figures S5, S6). The catalytic cycle of 2OG-dependent hydroxylases involves a step in which hydrogen abstraction from the substrate is followed by hydroxylation.[9] The abstracted hydrogen atom is usually observed to point towards the active-site iron center, as occurs in the complex of BBOX with NOG and GBB. In the BBOX **1** NOG Ni^II^ complex, the equatorial H3 (pro-*R*) atom of **1** also points towards the metal, in an orientation consistent with its abstraction in the catalytic cycle to give (3*R*)-hydroxy-(4*S*)-*N*,*N*-dimethyl-piperidine-4-carboxylic acid (**2**).

**Figure 2:**
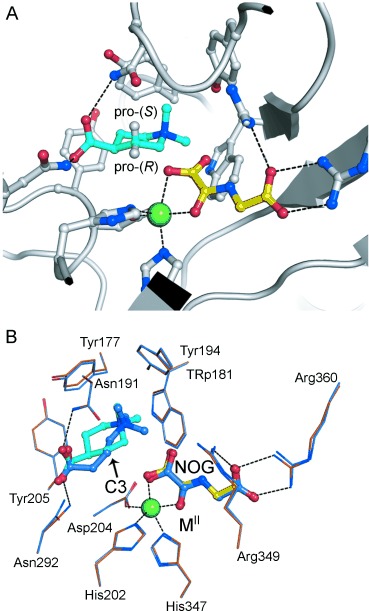
A crystal structure of BBOX complexed with *N*,*N*-dimethyl piperidine (1), Ni^II^ (as a substitute for Fe^II^), and NOG (*N*-oxyalyl glycine, a non-reactive 2OG mimetic). A) The view from the active site of the BBOX 1 Ni^II^ NOG complex (PDB ID: 4CWD) reveals that 1 adopts a chair conformation, with its pro(*R*) hydrogen atom, which is hydroxylated, pointing towards the metal. B) An overlay of the active sites of BBOX complexed with GBB, Zn^II^, and NOG (blue, PDB ID: 3O2G) or with 1, Ni^II^, and NOG (orange, PDB ID: 4CWD) reveal similar positions of the active-site residues and NOG. The carboxylate groups of GBB and 1 are similarly positioned, both interacting with the side chains of Asn191 and Asn292. The quaternary ammonium groups of both GBB and 1 occupy an identical aromatic cage formed by Tyr177, Tyr194, Tyr205, and Trp181. M=metal. Note: the carbons that undergo hydroxylation (C3 in both GBB and 1) are near identically positioned relative to the metal ion in both structures.

The results for the BBOX-catalyzed oxidation of *N*,*N*-dimethyl-piperidine-4-carboxylic acid (**1**) demonstrate the potential of BBOX for the desymmetrization of cyclic achiral compounds to produce a product with two stereocenters. We then examined the potential of BBOX to catalyze the hydroxylation of a piperidine-4-carboxylic acid alkylated with two different groups, specifically *N*,*N*-ethylmethyl-piperidine-4-carboxylic acid (**3**). Alkylation of the appropriate *N*-alkyl-piperidine-4-carboxylic acid gave **3** as a mixture of *cis*-*N*,*N*-ethylmethyl-piperidine-4-carboxylic acid (**3 a**) and *trans*-*N*,*N*-ethylmethyl-piperidine-4-carboxylic acid (**3 b**), with preferential ‘axial’ alkylation of the mono-alkylated intermediates observed in the synthesis of (**3 a**)/(**3 b**) [Figures S7, S8 and Scheme S1]. Reaction of the mixture of *cis*-**3 a** and *trans*-**3 b** with BBOX led to the preferential conversion of the *cis*-**3 a** isomer to give a single product (Figure S9), which could be assigned as (3*R*)-hydroxy-(1*R*)-*N*,*N*-dimethyl-piperidine-(4*S*)-carboxylic acid (**4 a**) with the aid of NMR spectroscopy (Figures S10–S12) and by analogy to the protein crystallography analysis of BBOX in complex with **1**. Initial studies on the hydroxylation rate revealed that, as with **1**, the hydroxylation of **3 a** to give **4 a** was two times slower than that of GBB, but 2OG decarboxylation remained tightly coupled to the hydroxylation (Figure S13 and Table S1 in the Supporting Information).

These results reveal the potential of non-heme oxygenases for the desymmetrisation of achiral compounds with the concomitant introduction of multiple stereocentres, as well as for kinetic resolution. The results also show limitations in the range of substrates accepted, but these might be addressed through engineering of the naturally occurring enzymes. Given the breadth of their reactions, it would thus seem that non-heme oxygenases, and in particular 2OG oxygenases, have considerable potential both for the production of chiral starting materials for high-value chemical production and for the late-stage oxidations of bioactive compounds. The latter is exemplified in the present work by the stereoselective oxidation of *N*,*N*-dialkyl piperidine-4-carboxylic acids.
