# Supplementary material for: Oxygenase-Catalyzed Desymmetrization of N,N-Dialkyl-piperidine-4-carboxylic Acids
Source: Angew Chem Int Ed Engl. 2014 Aug 27;53(41):10925–7. doi: 10.1002/anie.201406125 (PMC4497603; doi:10.1002/anie.201406125)
Supplement: Supplementary file 1 [file anie0053-10925-sd1.pdf]

Angewandte Chemie
Eine Zeitschrift der Gesellschaft Deutscher Chemiker

Supporting Information

© Wiley-VCH 2014

69451 Weinheim, Germany

Oxygenase-Catalyzed Desymmetrization of *N*,*N*-Dialkyl-piperidine-4-carboxylic Acids**

*Anna M. Rydzik, Ivanhoe K. H. Leung, Grazyna T. Kochan, Michael A. McDonough, Timothy D. W. Claridge, and Christopher J. Schofield**

anie_201406125_sm_miscellaneous_information.pdf

Contents

Initial MS screening 2

Further NMR assignments of BBOX catalysed hydroxylation of (1) 3

Crystallographical analyses 4

Conformational assignement of *N*,*N*-dimethyl isonipecotic acid 5

Preparation of *N*,*N*-ethylmethyl-piperidine-4-carboxylic acid (3) 6

Assignemnt of *N*,*N*-ethylmethyl-piperidine-4-carboxylic acid isomers 7

Assignments of product of BBOX catalysed hydroxylation of *N*,*N*-ethylmethyl-piperidine-4-carboxylic acid (3) 9

Kinetic analyses 13

Experimental 15

- Enzyme production 15
- MS methods 15
  - Initial MS screening 15
  - LC-MS method 15
- NMR 16
  - Hydroxylation product assignment 16
  - Time course assays 16
- Kinetic parameters measurment 16
- Crystallography 17
- Synthesis 19
  - General procedure for the synthesis of cyclic GBB analogues 19

References 30

Initial MS screening

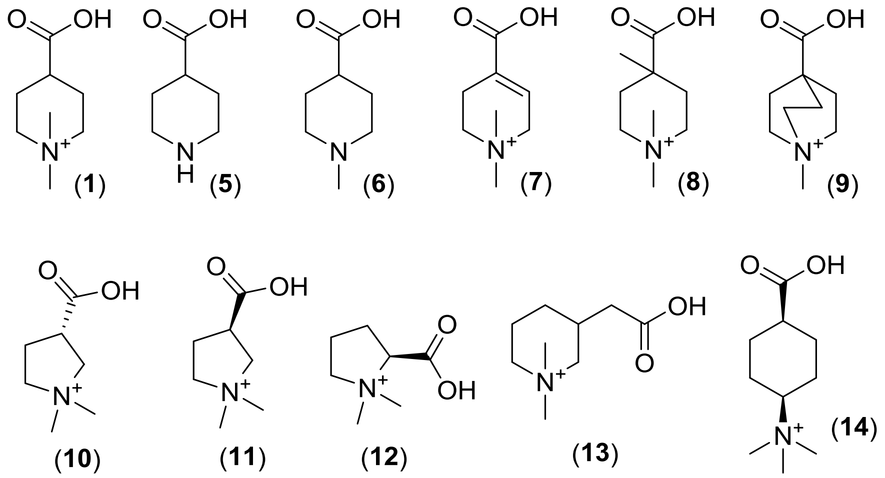

Figure S1. The set of cyclic GBB analogues used in initial substrate screening with BBOX. Only (**1**) was observed to be hydroxylated. The results imply that BBOX requires a quaternary ammonium centre for reactivity, since neither piperidine-4-carboxylic acid (**5**) nor *N*-methyl-piperidine-4-carboxylic acid (**6**) are substrates. Analogues with additional modifications in the piperidine ring (e.g. unsaturated analogue (**7**), or methylated analogue (**8**)) were not substrates. BBOX has an apparent requirement for a C-4 carboxylate, since (**13**) with a C-3 carboxylate was not hydroxylated. The 5-membered ring analogues were not hydroxylated.

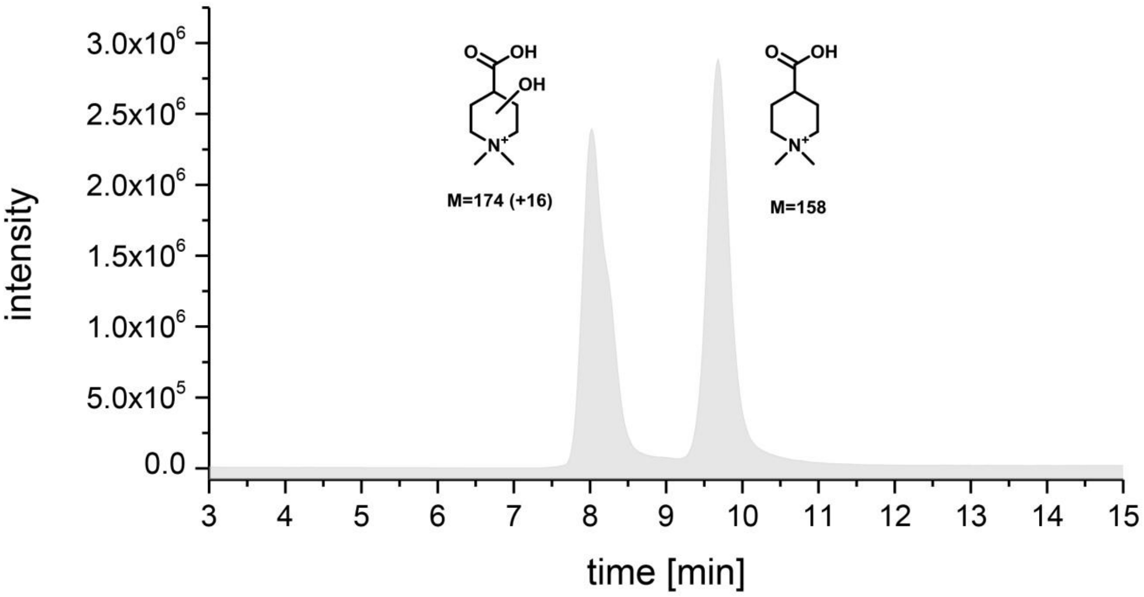

Figure S2. LC-MS chromatogram of the reaction of BBOX with *N,N*-dimethylisonipecotic acid (1). Spectra were recorded in mass range 120-300 Da (scan mode), single ion mode used in parallel to monitor masses: 158 (substrate, retention time = 9.68 min) and 174 (product, retention time = 8.02 min).

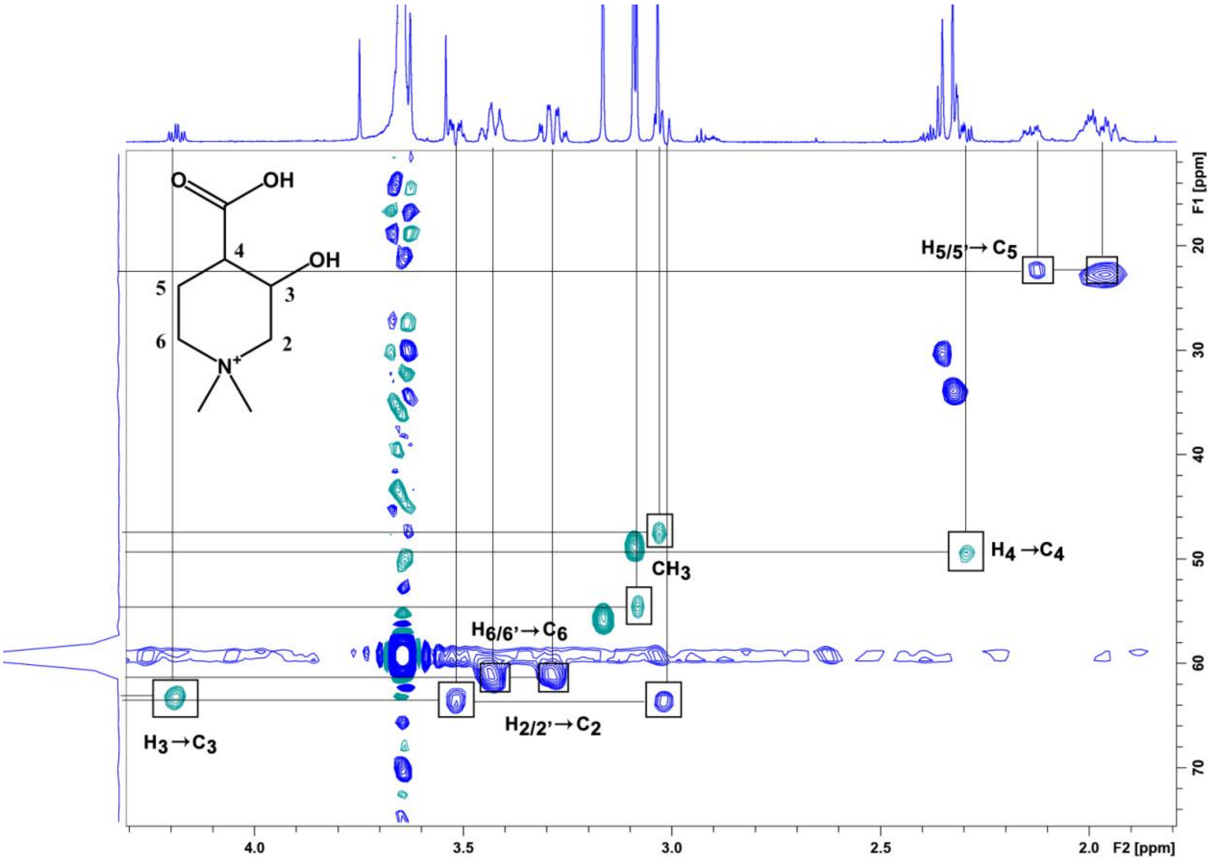

Figure S3. Further NMR assignments of BBOX catalysed hydroxylation of (1). Multiplicity edited ^{1}H-^{13}C HSQC spectra of *N*,*N* – dimethyl isonipecotic acid (**1**) reaction with BBOX. ^{13}C NMR (from HSQC signals) (176 MHz, D_2O) δ ppm 63.7 (C_2), 63.4 (C_3), 61.8 (C_6), 54.7 (CH_3), 49.5 (C_4), 47.5 (CH_3), 22.52 (C_5). ^{1}H NMR (700 MHz, D_2O) δ ppm 4.18 (1H, td, *J* = 10.8, 4.7 Hz, H_3), 3.54-3.49 (1H, m, H_2), 3.47-3.42 (1H, m, H_{6eq}), 3.33-3.28 (1H, m, H_{6ax}), 3.16 (3H, s, CH_3), 3.09 (3H, s, CH_3), 3.04-3.00 (1H, m, $H_{2'}$), 2.30 (1H, ddd, *J* = 12.4, 10.5, 4.7 Hz, H_4), 2.17-2.11 (1H, m, H_5), 1.97 (1H, m, $H_{5'}$).

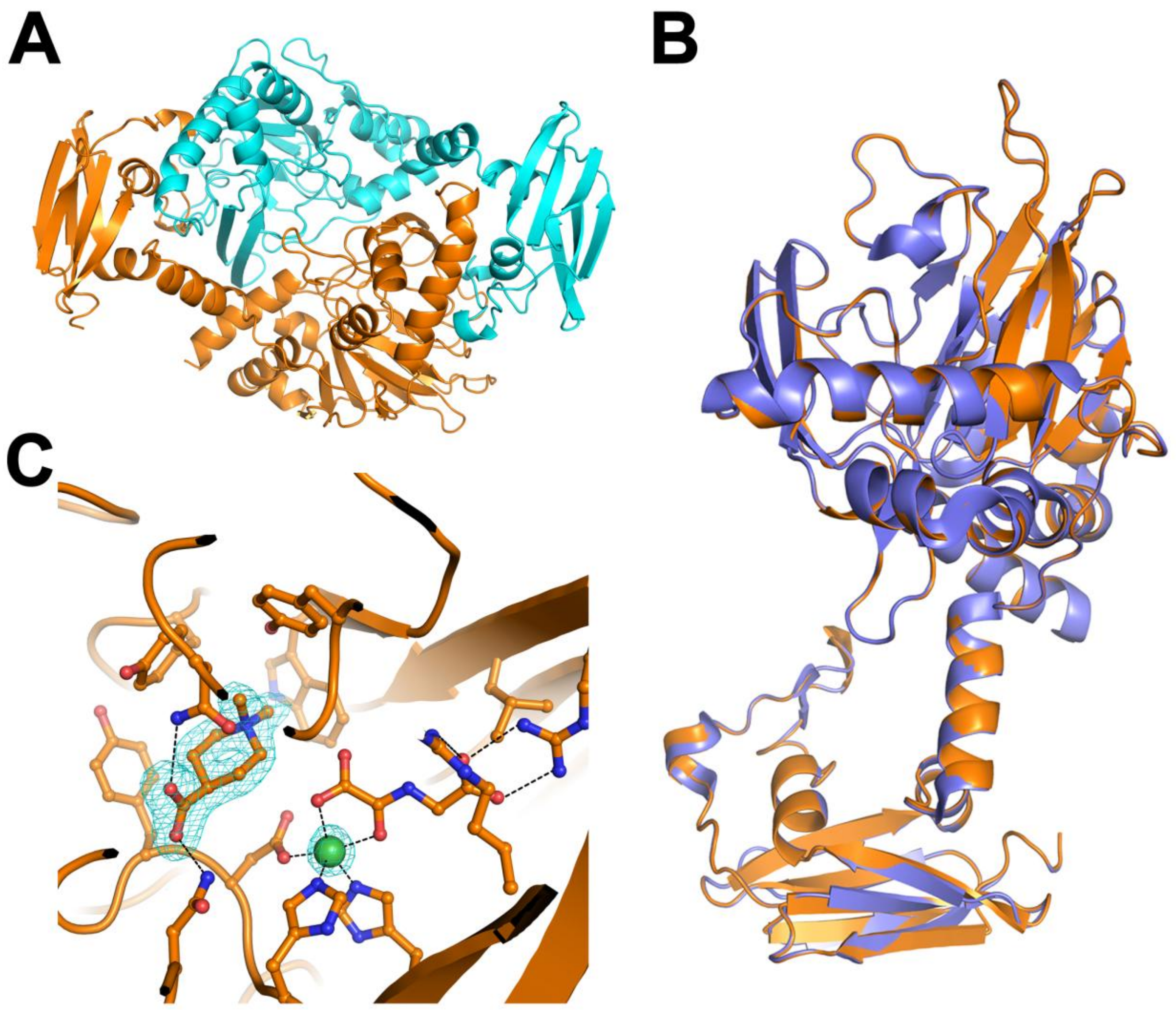

Figure S4. The crystal structure of human BBOX in complex with N,N-dimethylisonipecotic acid (**1**), NOG and Ni(II) is similar to that of the BBOX.GBB.NOG.Zn(II) complex[1]. (A) – View from the overall structure of the BBOX.(1).NOG.Ni(II) complex dimer (PDB id: 4CWD). (B) – Overlay of a structure of the BBOX.GBB.NOG.Zn(II) complex monomer (PDB id: 3O2G, blue) and BBOX.(1).NOG.Zn(II) complex monomer (PDB id: 4CWD, orange) reveal structural similarity. (C) – View from the active site of BBOX.(1).NOG.Zn(II) complex with electron density maps (OMIT Fo-Fc map contoured to 3σ).

Conformational assignement of *N,N*-dimethyl isonipecotic acid

The ^{1}H NMR spectrum of (**1**) in D_2O at room temperature displayed only one distinctive set of peaks, likely corresponding to a single conformer (Fig. S5). The position of H4, adjacent to the carboxylic group, was assigned as axial, based on its coupling constant analysis (J_{aa} = 11.2 Hz, J_{ae} = 4.6 Hz). The overall signal pattern was characteristic for a chair conformation (The slight phase distortions seen for the multiplets arises from the excitation sculpting water suppression scheme employed). The identity of signals was further confirmed by assignment of ^{13}C NMR shifts for (**1**) using ^{1}H-^{13}C HSQC spectra (Fig. S6).

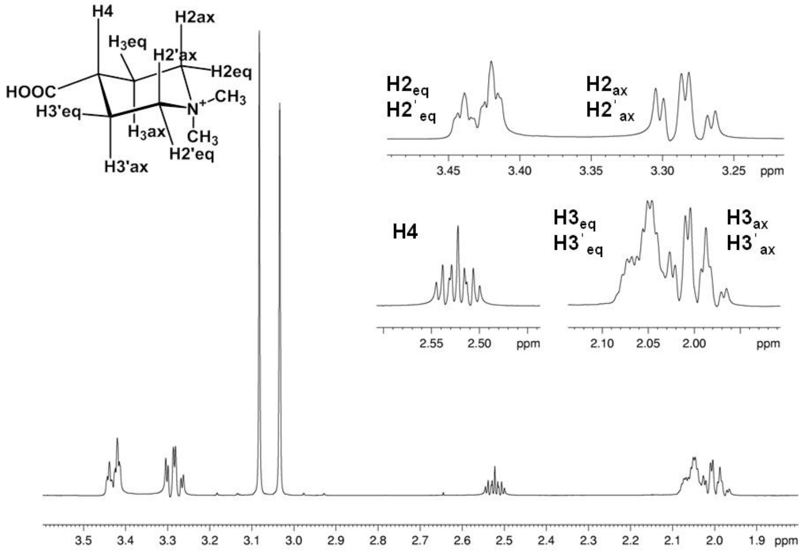

Figure S5. Conformation of *N*,*N*-dimethyl isonipecotic acid (**1**) as assigned by ^{1}H NMR. ^{1}H NMR (700 MHz, D_2O) δ ppm 3.43 (2H, dt, J = 13.1, 3.7 Hz, H_{2eq}, $H_{2'eq}$), 3.28 (2H, td, J = 12.7, 3.6 Hz, H_{2ax}, $H_{2'ax}$), 3.08 (3H, s, CH_3), 3.03 (3H, s, CH_3), 2.64 (1H, tt, J = 11.2, 4.6 Hz, H_4), 2.09-2.02 (2H, m, H_{3eq}, $H_{3'eq}$), 2.02-1.96 (2H, m, H_{3ax}, $H_{3'ax}$).

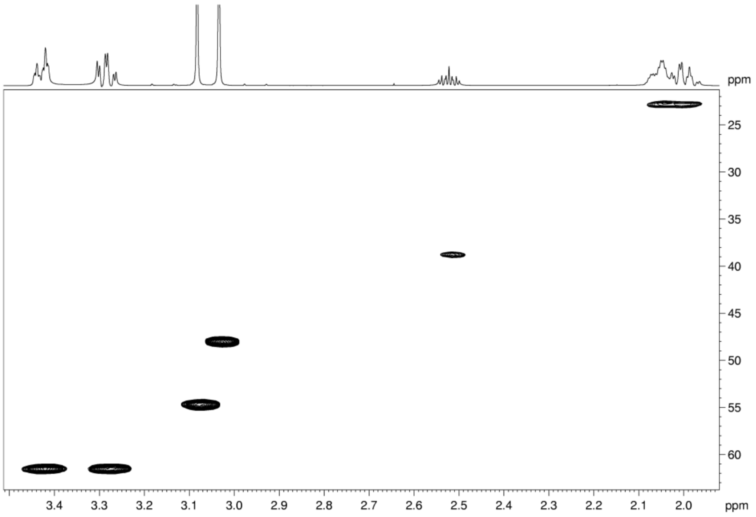

Figure S6. Conformation of *N*,*N*-dimethyl isonipecotic acid (**1**) as assigned by ^{1}H-^{13}C HSQC. ^{13}C NMR (from HSQC signals) (176 MHz, D_2O) δ ppm 61.5 (C_2, $C_{2'}$), 54.7 (CH_3), 47.9 (CH_3), 38.7 (C_4), 22.7 (C_3, $C_{3'}$).

Preparation of *N,N*-ethylmethyl-piperidine-4-carboxylic acid (3)

N,N-Ethylmethyl-piperidine-4-carboxylic acid (**3**) was prepared from commercially available *N*-methyl isonipecotic acid (**6**) and *N*-ethyl isonipecotic acid (**15**) via alkylation with requisite methyl/ethyl iodide at room temperature. In both cases, the resulting product was a mixture of two isomers (Scheme S1). Preferential 'axial' alkylation was observed under the tested reaction conditions.

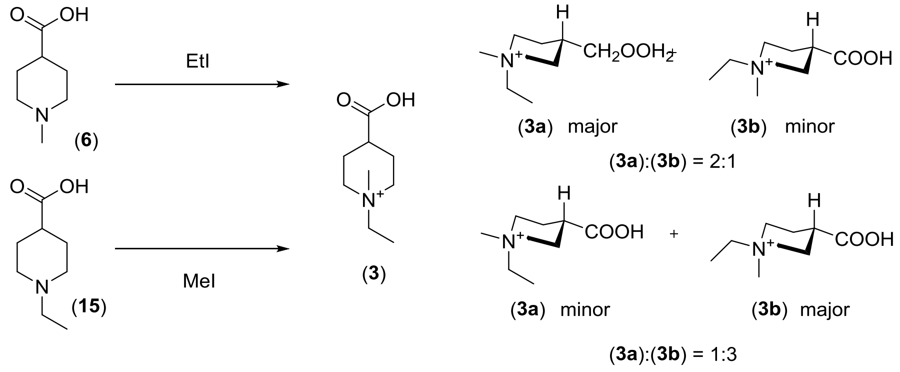

Scheme S1. Synthesis of *N,N*-ethylmethyl-piperidine-4-carboxylic acid (**3**). Analogue (**3**) was prepared by alkylation of (**6**) or (**15**) by ethyl and methyl iodide (3 eq.), respectively. Reaction was conducted in methanol in the presence of K_2CO_3 (3 eq.) in methanol at room temperature.

Assignment of *N,N*-ethylmethyl-piperidine-4-carboxylic acid isomers

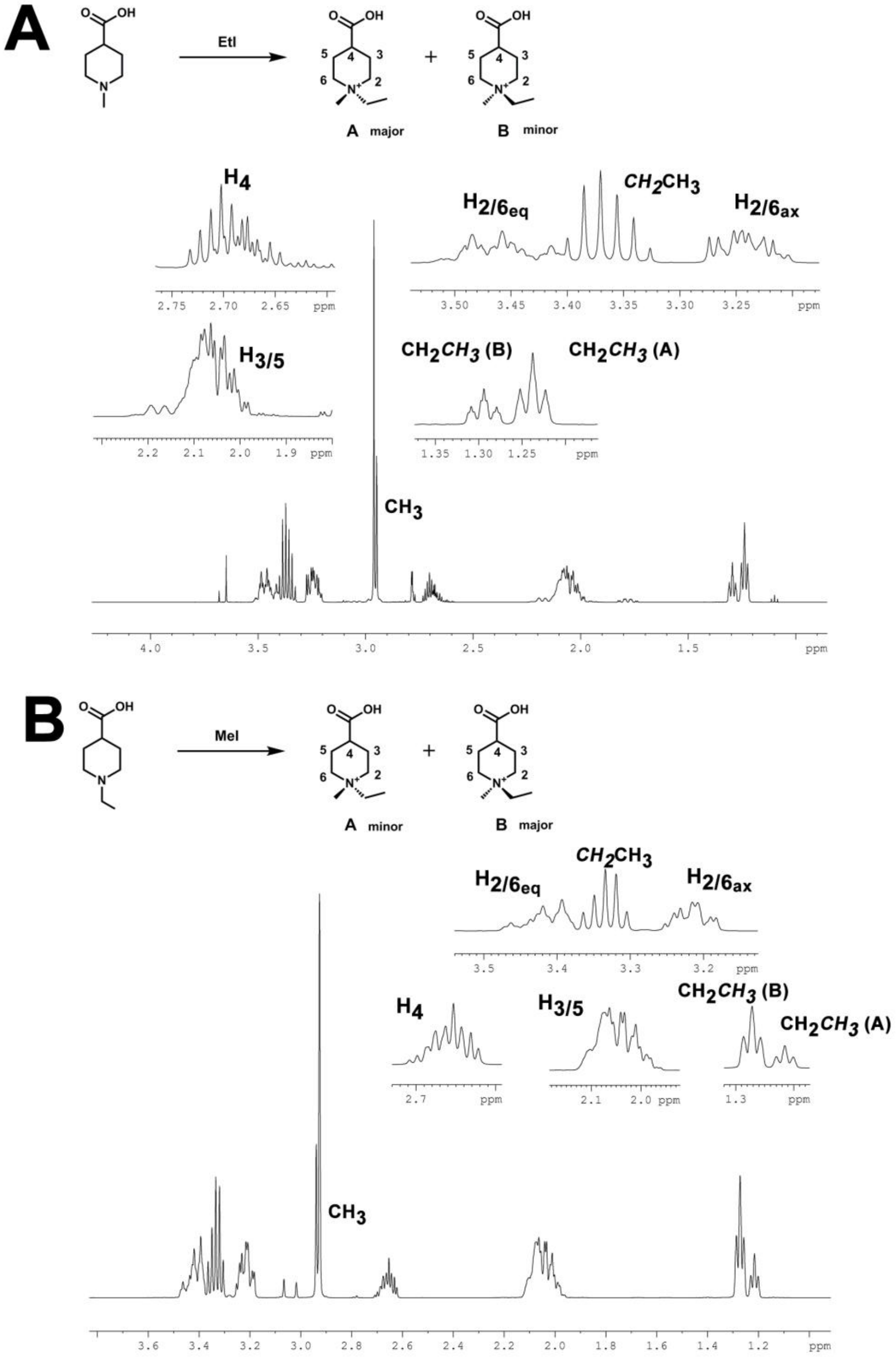

Figure S7. ^{1}H NMR spectra of *N,N*-ethylmethyl-piperidine-4-carboxylic acid (**3**) obtained by alkylation of *N*-methyl-piperidine-4-carboxylic acid (**6**) (top) or *N*-ethyl-piperidine-4-carboxylic acid (**15**) (bottom). *Isomer A*: ^{1}H NMR (700 Hz, D_2O) δ ppm 3.47 (2H, dt, *J* = 13.3, 4.4 Hz, H_{2eq}, H_{6eq}), 3.38 (2H, q, *J* = 7.5 Hz, CH_2CH_3), 3.24 (2H, td, *J* = 11.5, 3.8 Hz, H_{2ax}, H_{6ax}), 2.96 (3H, s, CH_3), 2.63 (1H, tt, *J* = 10.2, 5.1 Hz, H_4), 2.12-1.99 (4H, m, H_{3eq}, H_{5eq}, H_{3ax}, H_{5ax}), 1.25 (3H, t, J = 7.8, CH_2CH_3). *Isomer B*: ^{1}H NMR (700 Hz, D_2O) δ ppm 3.43 (2H, dt, *J* = 13.5, 3.8 Hz, H_{2eq}, H_{6eq}), 3.35 (2H, q, *J* = 7.5 Hz, CH_2CH_3), 3.22 (2H, td, *J* = 13.1, 3.6 Hz, H_{2ax}, H_{6ax}), 2.95 (3H, s, CH_3), 3.03 (3H, s, CH_3), 2.61 (1H, tt, *J* = 11.5, 4.8 Hz, H_4), 2.12-1.99 (4H, m, H_{3eq}, H_{5eq}, H_{3ax}, H_{5ax}), 1.30 (3H, t, J = 7.8, CH_2CH_3).

The methyl group position was assigned by nOe studies (Fig. S8). The H4 proton (i.e. adjacent to the carboxylate) was assigned to be axial in both isomers of (**3**) by the coupling constants pattern in the ^{1}H NMR spectra (isomer A: J_{aa} = 10.2 Hz, J_{ae} = 5.1 Hz; isomer B: J_{aa} = 11.5 Hz, J_{ae} = 4.8 Hz).

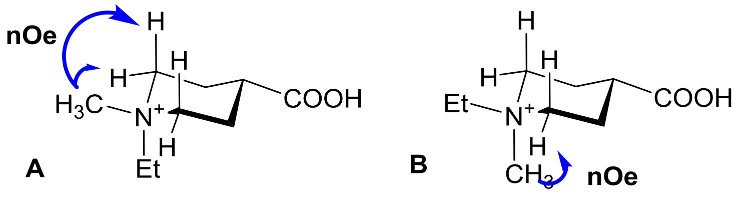

Figure S8. nOe correlations for isomers of *N,N*-ethylmethyl-piperidine-4-carboxylic acid (**3**).

Assignments of product of BBOX catalysed hydroxylation of *N,N*-ethylmethyl-piperidine-4-carboxylic acid (3)

Mixtures of isomers (**3a**) and (**3b**) (mixture 1: (**3a**):(**3b**) = 2:1, mixture 2: (**3a**):(**3b**) = 1:3) were subjected to BBOX catalysed oxidation. MS based assay indicated the formation of new species with a mass shift of +16, corresponding to a single hydroxylation (Fig. S9).

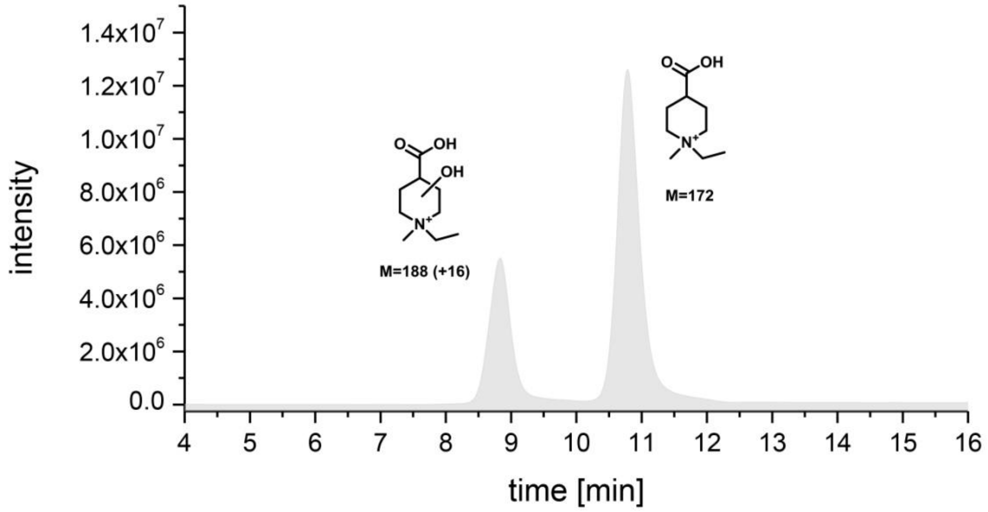

Figure S9. LC-MS chromatogram of the reaction of BBOX with *N*-methyl-*N*-ethylisonipecotic acid (**3**) (used as a mixture of isomers (**3a**) and (**3b**) – mixture 1). Spectra were recorded in mass range 120-300 Da (scan mode), single ion mode used in parallel to monitor masses: 172 (substrate, retention time = 10.79 min) and 188 (product, retention time = 8.86 min).

In case of both Mixture 1 and 2 a formation of new peaks in the ^{1}H NMR spectra was observed. In both cases, the presence of two new peaks, characteristic for axial protons adjacent to hydroxyl and carboxylate moiety was observed (4.17 and 2.32 ppm, respectively) (Fig. S10 and S11). In case of mixture 1, where isomer (**3a**) was the major component only one set of new peaks was observed, indicating that (**3a**) is the preferred substrate. Interestingly, for mixture 2, where isomer (**3b**) was the major component, the presence of second set of product peaks was detected; the major product was the same as that observed with Mixture 1 (Fig. S10 and S11). The result suggest that (**3b**) is hydroxylated by BBOX, but is a less preferred substrate than (**3a**) (even though (**3b**) was in excess in Mixture 2, the major product arises from hydroxylation of the minor component (**3a**)). Due to the low level of (**3b**) hydroxylation and signal overlap the stereochemistry of minor product has not been assigned with confidence.

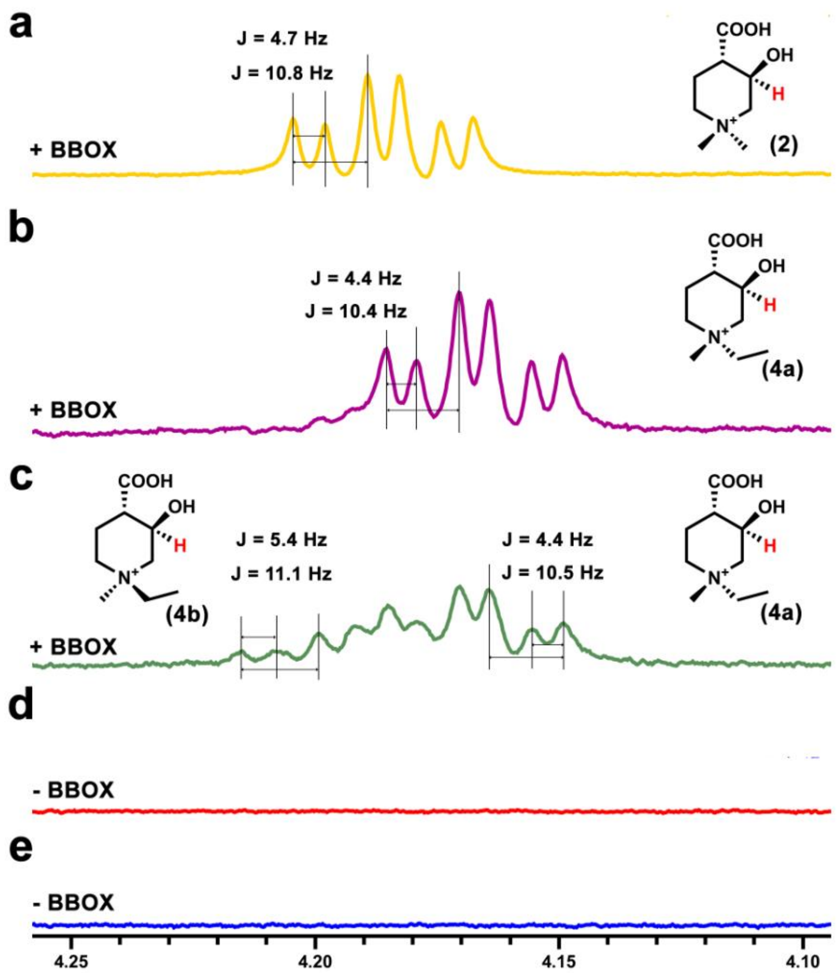

Figure S10. Overlay of the 4.10-4.25 ppm region of the ^{1}H NMR spectra of cyclic analogue reaction mixtures. (**a**) H_3 peak of BBOX catalysed oxidation of (**1**). H_3 was assigned to be in axial position (J_{aa} = 10.8 Hz × 2, J_{ae} = 4.7 Hz). (**b**) When a mixture of (**3a**):(**3b**) = 2:1 was subjected to BBOX, H_3 was also assigned to be axial (J_{aa} = 10.4 Hz × 2, J_{ae} = 4.4 Hz). Only one hydroxylation product was observed. (**c**) When a mixture of (**3a**):(**3b**) = 1:3 was subjected to BBOX, two peaks corresponding to H_3 were observed, both assigned as axial protons (major: J_{aa} = 10.5 Hz × 2, J_{ae} = 4.4 Hz, minor: J_{aa} = 11.1 Hz × 2, J_{ae} = 5.4 Hz). The major product is same as that product obtained for the mixture in (b) and corresponds to the hydroxylation product of (**3a**) to give (**4a**). The minor product likely corresponds to hydroxylation of (**3b**) to give (**4b**), which was the major component of substrate mixture used in (c). (**d**) Reaction mixture containing substrate mixture as in (b), before addition of BBOX. (**e**) Reaction mixture containing substrate mixture as in (c) before addition of BBOX.

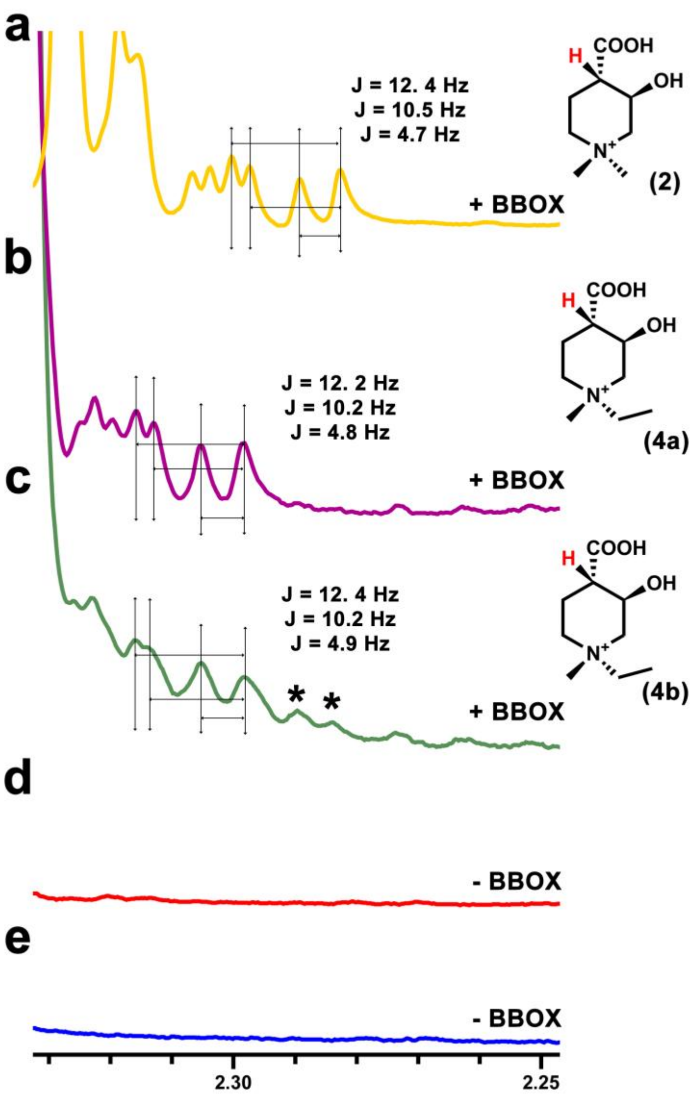

Figure S11. Overlay of the 2.25-2.32 ppm region of ^{1}H NMR spectra of cyclic analogues reaction mixtures. (**a**) H_4 peak of BBOX catalysed oxidation of (**1**). H_4 was assigned to be in axial position (J_{aa} = 12.4 Hz, J_{aa} = 10.5 Hz, J_{ae} = 4.7 Hz). (**b**) When a mixture of (**3a**):(**3b**) = 2:1 was subjected to BBOX oxidation, H_4 of the product was assigned as axial (J_{aa} = 12.2 Hz, J_{aa} = 10.2 Hz, J_{ae} = 4.8 Hz). Only one hydroxylation product was observed. (**c**) When a mixture of (**3a**):(**3b**) = 1:3 subjected to BBOX a mixture of signals corresponding to H_4 was observed, the major signal assigned as corresponding to an axial proton (J_{aa} = 12.4 Hz, J_{aa} = 10.2 Hz, J_{ae} = 4.8 Hz), identical that signal obtained for mixture as in (b). The *J* values of the H_4 signal of the minor product could not be assigned due to signal overlap. Starred peaks likely correspond to the minor product. (**d**) Reaction mixture containing substrate mixture as in (b), before addition of BBOX. (e) Reaction mixture containing substrate mixture as in (c), before addition of BBOX. The multiplets observed are partially masked by other signals in the spectra.

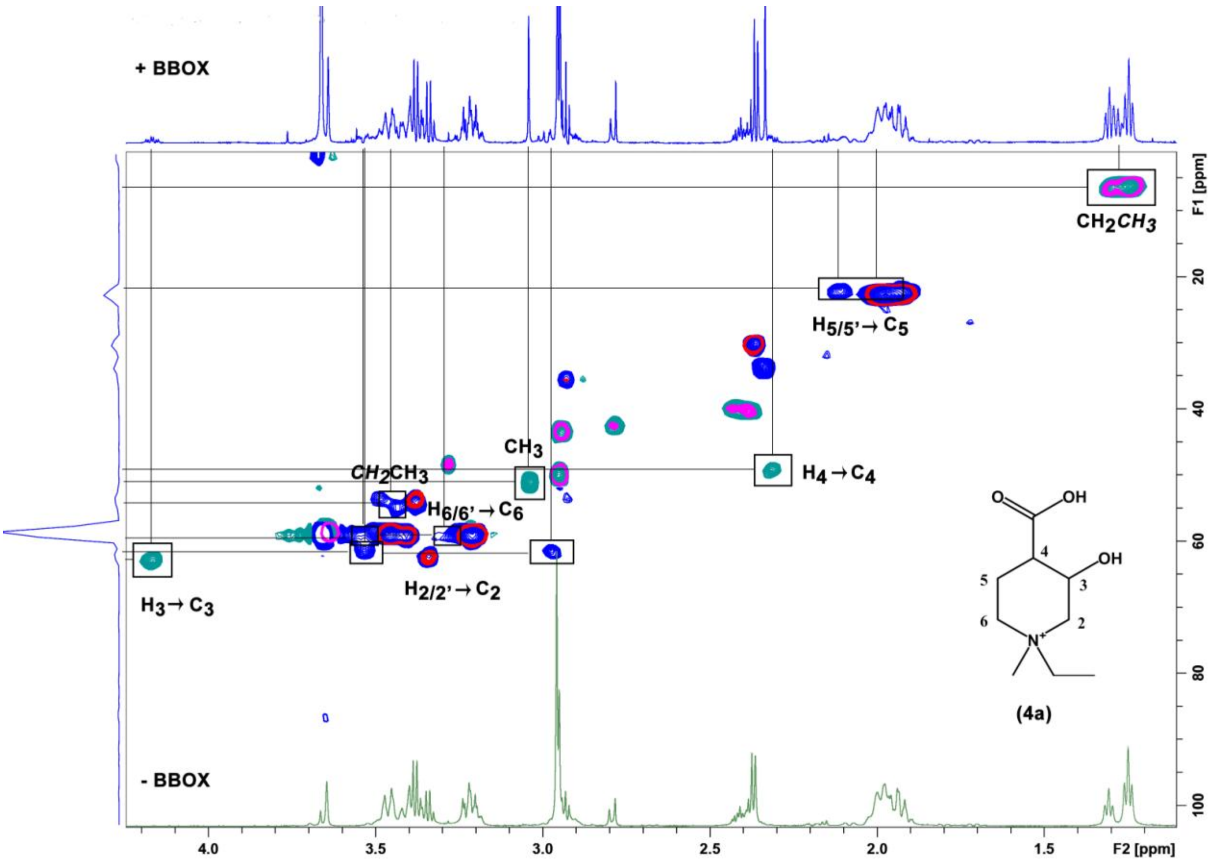

Figure S12. Multiplicity edited ^{1}H-^{13}C HSQC assignments of product of BBOX catalysed hydroxylation of *N*,*N*-ethylmethyl-piperidine-4-carboxylic acid (**3**) (from the mixture where (**3a**):(**3b**) = 2:1). The picture represents an overlay of the ^{1}H-^{13}C HSQC spectra of reaction mixture before the addition of BBOX (red/pink) and after a 24 h incubation with 8 µM BBOX (blue/green).

Kinetic analyses

Table S1. Kinetic parameters of BBOX catalysed hydroxylation of GBB and cyclic GBB analogues (**1**) and (**3a**).

Kinetic parameter	GBB	(**1**)	(**3a**)
Initial rate of hydroxylation [μM/s]	0.130	0.059	0.041
Initial rate of succinate formation [μM/s]	0.162	0.078	0.077
K_M [μM]	4*	40	-
V_{max} [μM/s]	0.041*	0.054	-
k_{cat} [1/s]	0.83*	0.14	-
K_I [μM]	25*	621	-
yield**	78%	32%	27%

* Data from [2]. **yield is given as a percentage conversion of substrate into hydroxylated product (as calculated by ^{1}H NMR) after 20 min of the reaction.

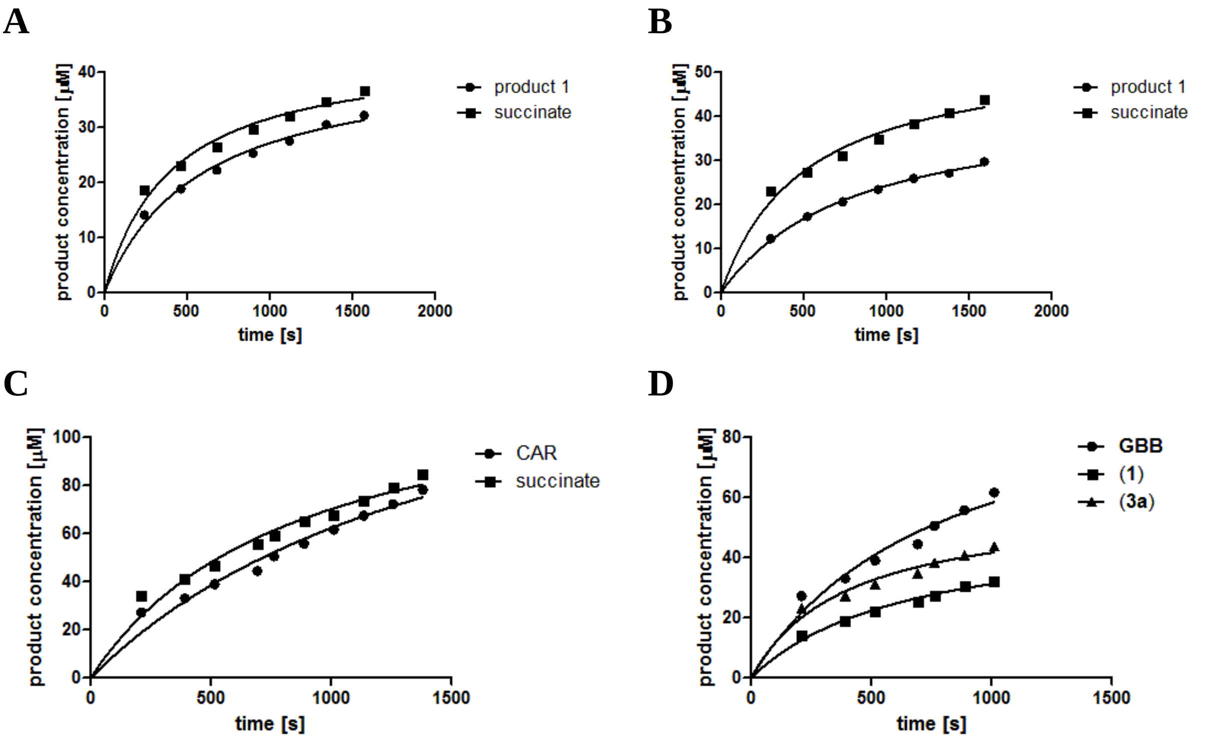

Figure S13. Time course of BBOX catalysed oxidation of GBB analogues. (A) Time course of BBOX catalysed oxidation of (**1**) and succinate formation. (B) Time course of BBOX catalysed oxidation of (**3a**) and succinate formation. (C) Time course of BBOX catalysed oxidation of GBB and succinate formation. (D) Comparison of time courses of BBOX catalysed oxidation of GBB and cyclic GBB analogues (**1**) and (**3a**). Conditions are as described in the SI: Experimental section – Time course analyses (p. S16).

A **B**

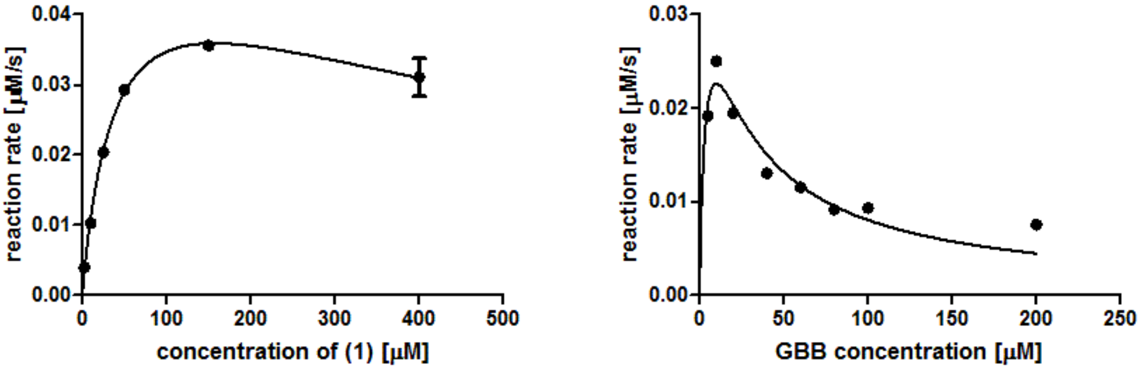

Figure S14. Dependence of the initial reaction rate on the concentration of cyclic GBB analogue (**1**).

Experimental

Enzyme production

Recombinant human BBOX was produced and purified as described[1].

MS methods

Initial MS screening

Initial screens for BBOX substrates were performed on Waters LCT Premier Instrument, employing Electron impact Chemical Ionisation, fitted with time of flight (ToF) analyser. Samples were measured using direct injection (no column attached) and analysed for the presence of a +16 peak.

Enzymatic assays were run in the following conditions: 100 µM substrate, 1 mM 2-oxoglutarate disodium salt, 100 µM Fe(II) ($Fe(NH_4)_2(SO_4)_2$ salt, solution prepared fresh before experiment from concentrated stock in 20 mM HCl), 200 mM KCl, 500 µM ascorbic acid sodium salt, 1 µM enzyme, buffer: 50 mM phosphate pH 7.0, final volume 200 µL. Each assay contained samples run with BBOX and a control with no enzyme added.

LC-MS method

Chromatographic separation of the GBB analogues was performed using mixed mode chromatography. Chromatographic separation was performed using an Aquity UPLC system (Waters). Column: PrimeSep 200 mixed mode, 2.1×250mm, particles 5µm (SIELC, Prospect Heights, US). Mobile phase: Solvent A – 9:1 H_2O-acetonitrile mixture, 0.05% formic acid, solvent B – 8:2 H_2O-acetonitrile mixture, 0.2% formic acid. Gradient: Linear gradient from 0% to 100% B in 25 min, column reconditioning: 25-26 min from 0% to 100% A, 26-30 min 100% A. Flow rate: 0.3 mL/min. Injection volume 10 µL. Detection was performed using a Waters Quattro Micro instrument (triple quadrupole MS, electrospray ionisation, positive ion mode). The single ion mode was used and scan mode was running in parallel as a control. The reaction samples were prepared as described in the method for initial MS screening.

NMR

NMR assays were performed using a Bruker AVIII 700 instrument equipped with an inverse TCI cryoprobe using 3 mm MATCH tubes. Pulses were calibrated using single-pulse nutation method (Bruker pulsecal routine). Water suppression was achieved using the excitation sculpting method.

Hydroxylation product assignment

Hydroxylation reactions were performed using the following conditions: 0.8 mM 2OG (disodium salt), 80 mM KCl, 50 µM Fe(II) (used as an $(NH_4)_2Fe(SO_4)_2$ salt and prepared freshly before reaction start), 0.2 mM GBB analogue in 50 mM TRIS-d_{11} pH 7.5 in D_2O. Reaction was started by addition of human BBOX to the final concentration of 8 µM. Mixture was incubated overnight and then products were assigned using ^{1}H and multiplicity edited ^{1}H-^{13}C HSQC NMR.

Time course assays

Time course assays were run using the following conditions: GBB/GBB analogue 100 µM, 2OG 0.5 mM (disodium salt), ascorbate 0.5 mM (mono potassium salt), KCl 200 mM, Fe(II) 50 µM (used as an $(NH_4)_2Fe(SO_4)_2$ salt and prepared freshly before reaction start) in 50 mM Tris d_{11} pH 7.5, 10% D_2O. Reaction was initiated by the addition of human BBOX to the final concentration of 400 nM. First spectrum was measured after 210 sec from the reaction start. Each spectrum was measured with 16 transients.

Kinetic parameters measurment

Reactions include 400 nM BBOX enzyme, 50 µM Fe(II), 312.5 µM 2OG, 500 µM ascorbate and varying concentrations of substrate (2.5 µM, 10 µM, 25 µM, 50 µM, 150 µM, 400 µM). The buffer includes 50 mM Tris-D11, 80 mM KCl, 0.02% NaN_3 dissolved in 90% H_2O and 10% D_2O. The pH was adjusted to pH 7.5. 2OG oxidation into succinate was monitored for six data points between 300 seconds and 740 seconds and initial rates were obtained by fitting a linear equation to the data.

Crystallography

Crystallization was performed in 24-well plates; hanging drops were equilibrated against 400 µl of reservoir solution. Drops consisted of 1 µl reservoir solution and 1 µl protein solution. The reservoir solution contained: 0.2 M ammonium citrate, 1 mM $NiSO_4$, 4% 1,6-diaminohexane, 19% PEG 3350. Protein (20 mg/ml) was in TRIS buffer (50 mM pH 7.5) containing 200 mM NaCl and was supplemented with 5 mM *N*,*N*- dimethylisonipecotic acid (**1**) and 10 mM NOG (100 mM stock solutions in TRIS 50 mM, pH adjusted to 7.0) prior to crystallization. Crystals formed overnight.

Crystals were harvested in nylon loops and flash cooled in liquid nitrogen using 25% glycerol in mother liquor as a cryoprotectant. Data were collected on a single crystal in-house using a Rigaku FRE+ SuperBright X-ray diffractometer equipped with Osmic HF optics and a Saturn944+ CCD detector. Data were integrated and scaled using HKL3000[3]. The structure was solved by molecular replacement using PHASER[4] (search model PDB ID: 3O2G). Iterative cycles of model building in COOT[5] and refinement using PHENIX[6] proceeded until the converging R_{cryst} and R_{free} no longer decreased.

**Structural Complex**	**BBOX1:(1)**
**PDB ID**	**4CWD**
**Data Collection**	
Wavelength (Å)	1.5418
Temperature	100 K
Space group	*H*3 2
Cell dimensions	
a, *b*, *c* (Å)	106.52, 106.52, 205.35
α,β,γ (°)	90.00, 90.00, 120.00
Resolution range * (Å)	24.83 − 1.9 (1.97−1.9)
R_{merge}	0.17
$I/\sigma I$ *	11.8 (1.4)
Completeness * (%)	99.7 (100.0)
Redundancy *	10.3(10.2)
Wilson B ($Å^2$)	23.8
Number of unique reflections	35551
**Refinement**	
Resolution (Å)	1.9
R_{work} / R_{free}	0.167/0.206
No. atoms	3553
Protein	3112
Ligand/ion	441
B-factors ($Å^2$)	25.72
Protein	24.70
Solvent	33.50
R.m.s. deviations	
Bond lengths (Å)	0.006
Bond angles (°)	0.96
Ramachandran favoured (%)	98.5
Clash score	0.81

Synthesis

Isonipecotic acid, its derivatives and other chemicals were from Sigma-Aldrich and used without further purification. Solvents were from Aldrich at HPLC grade, and used without further purification. Deuterated solvents were from Sigma and Apollo Scientific Ltd. ^{1}H NMR spectra were recorded using Bruker AVANCE AV400 (400 MHz), Bruker AVII 500 MHz with ^{13}C cryoprobe, Bruker AVIII 700 with inverse TCI cryoprobe or. Signal positions were recorded in δ ppm with the abbreviations br s., s, d, t, q, and m denoting broad singlet, singlet, doublet, triplet, quartet and multiplet respectively. All NMR chemical shifts were referenced to residual solvent peaks. Coupling constants, *J*, are registered in Hz to a resolution of 0.5 Hz. All compounds used in screening were more that 90% pure by ^{1}H NMR. High Resolution (HR) mass spectrometry data (m/z) were obtained from a Bruker MicroTOF instrument using an ESI source and Time of Flight (TOF) analyzer. Values are reported as ratio of mass to charge in Daltons.

General procedure for the synthesis of cyclic GBB analogues

The appropriate amino acid (1 equiv.) was dissolved in methanol (5 mL) along with K_2CO_3 (4 equiv.), treated with an excess of iodomethane (5 equiv.) and stirred at room temperature for 24 h. The methanol was evaporated, and the resultant residue dissolved in water (1 mL) and acidified with concentrated HCl. The mixture was stirred for 1 h and then washed with diethyl ether. Water was then evaporated *in vacuo* and the resultant residue was purified by HPLC (preparative C-18 reverse phase column; gradient: 50% B in 15 min, where A – water, 0.05% formic acid, B – acetonitrile, 0.1% formic acid; fractions containing product identified using Evaporative Light Scattering Detection (ELSD). The desired fractions were combined and freeze-dried to yield the product as a highly hygroscopic solid. Due to low level of compound recovery after HPLC purification, some of the assays used crude products if pure by ^{1}H NMR (which contained compound and KCl mixture, concentration of organic sample was determined by ^{1}H NMR). Due to small amount of purified product IR spectra and optical rotation values are not reported. Melting points are not given due to highly hygroscopic nature of the obtained materials.

4-Carboxy-1,1-dimethylpiperidin-1-ium (1)

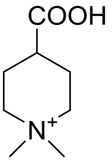

The trifluoroacetic acid (TFA) salt of desired product (11 mg, 0.04 mmol, 10%) was obtained starting from isonipecotic acid (50 mg, 0.39 mmol) by following the general procedure.

^{1}H NMR (700 MHz, D_2O) δ = 3.47 (dt, *J*=13.0, 3.0 Hz, 2 H), 3.33 (td, *J*=12.5, 3.5 Hz, 2 H), 3.12 (s, 3 H), 3.07 (s, 3 H), 2.67 (tt, *J*=10.5, 5.0 Hz, 1 H), 2.10 - 2.17 (m, 2 H), 2.01 - 2.10 (m, 2 H) ppm, ^{13}C NMR (126 MHz, D_2O) δ = 177.8, 61.4, 54.1, 48.1, 37.3, 22.3 ppm. HRMS (ESI-TOF) calcd for $C_8H_{16}NO_2^+$ [M^+]: 158.1176, found: 158.1168.

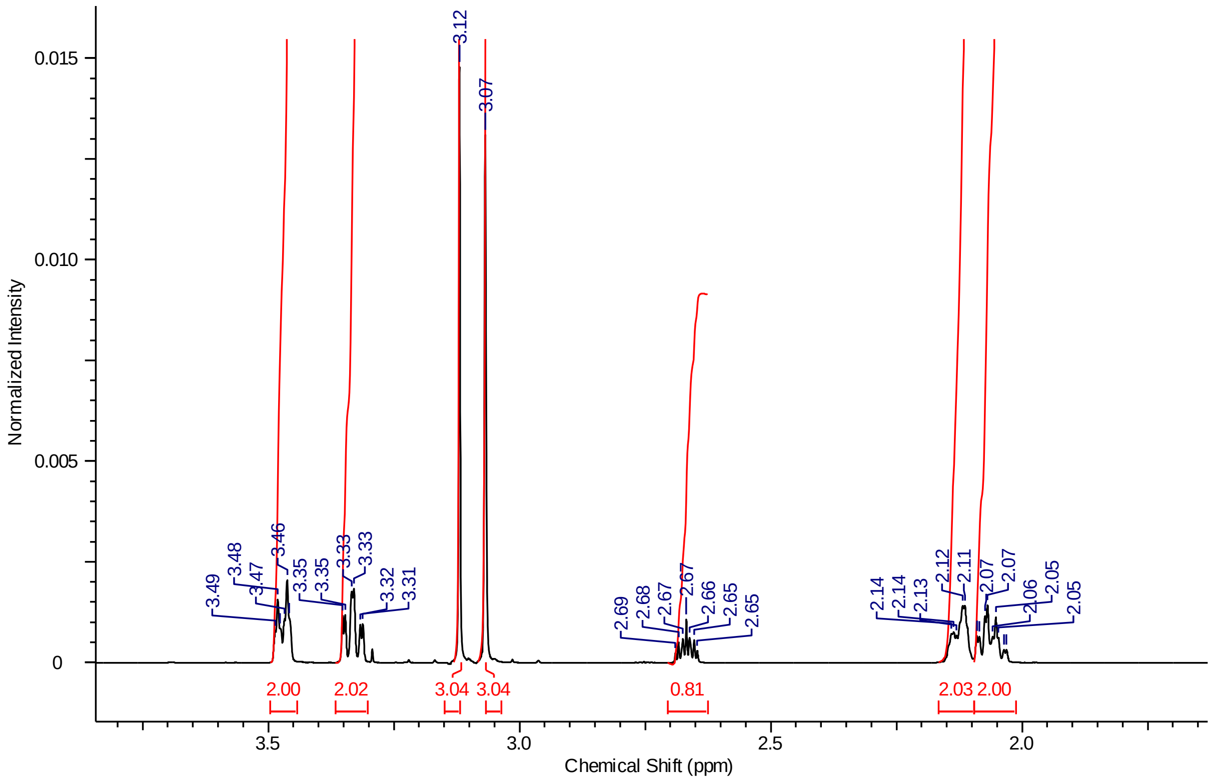

4-Carboxy-1-ethyl-1-methylpiperidin-1-ium (2)

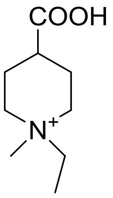

The trifluoroacetic acid (TFA) salt of desired product (9 mg, 0.03 mmol, 8%) was obtained starting from *N*-methylisonipecotic acid (50 mg, 0.39 mmol) or *N*-ethylisonipecotic acid (50 mg, 0.38 mmol) by following the general procedure. The compound was obtained as a mixture of conformers.

Isomer A: ^{1}H NMR (700 Hz, D_2O) δ ppm 3.47 (2H, dt, *J* = 13.3, 4.4 Hz, H_{2eq}, $H_{2'eq}$), 3.38 (2H, q, *J* = 7.5 Hz, CH_2CH_3), 3.24 (2H, td, *J* = 11.5, 3.8 Hz, H_{2ax}, $H_{2'ax}$), 2.96 (3H, s, CH_3), 2.63 (1H, tt, *J* = 10.2, 5.1 Hz, H_4), 2.12-1.99 (4H, m, H_{3eq}, $H_{3'eq}$, H_{3ax}, $H_{3'ax}$), 1.25 (3H, t, J = 7.8, CH_2CH_3). ^{13}C NMR (176 MHz) δ ppm 58.7, 54.7, 49.7, 36.9, 21.5, 6.43. Isomer B: ^{1}H NMR (700 Hz, D_2O) δ ppm 3.43 (2H, dt, *J* = 13.5, 3.8 Hz, H_{2eq}, $H_{2'eq}$), 3.35 (2H, q, *J* = 7.5 Hz, CH_2CH_3), 3.22 (2H, td, *J* = 13.1, 3.6 Hz, H_{2ax}, $H_{2'ax}$), 2.95 (3H, s, CH_3), 3.03 (3H, s, CH_3), 2.61 (1H, tt, *J* = 11.5, 4.8 Hz, H_4), 2.12-1.99 (4H, m, H_{3eq}, $H_{3'eq}$, H_{3ax}, $H_{3'ax}$), 1.30 (3H, t, J = 7.8, CH_2CH_3). ^{13}C NMR (176 MHz) δ ppm 62.5, 58.8, 44.1, 37.4, 21.5, 6.68. HRMS (ESI-TOF) calcd for $C_9H_{18}NO_2^+$ [M^+]: 172.1332, found: 172.1338.

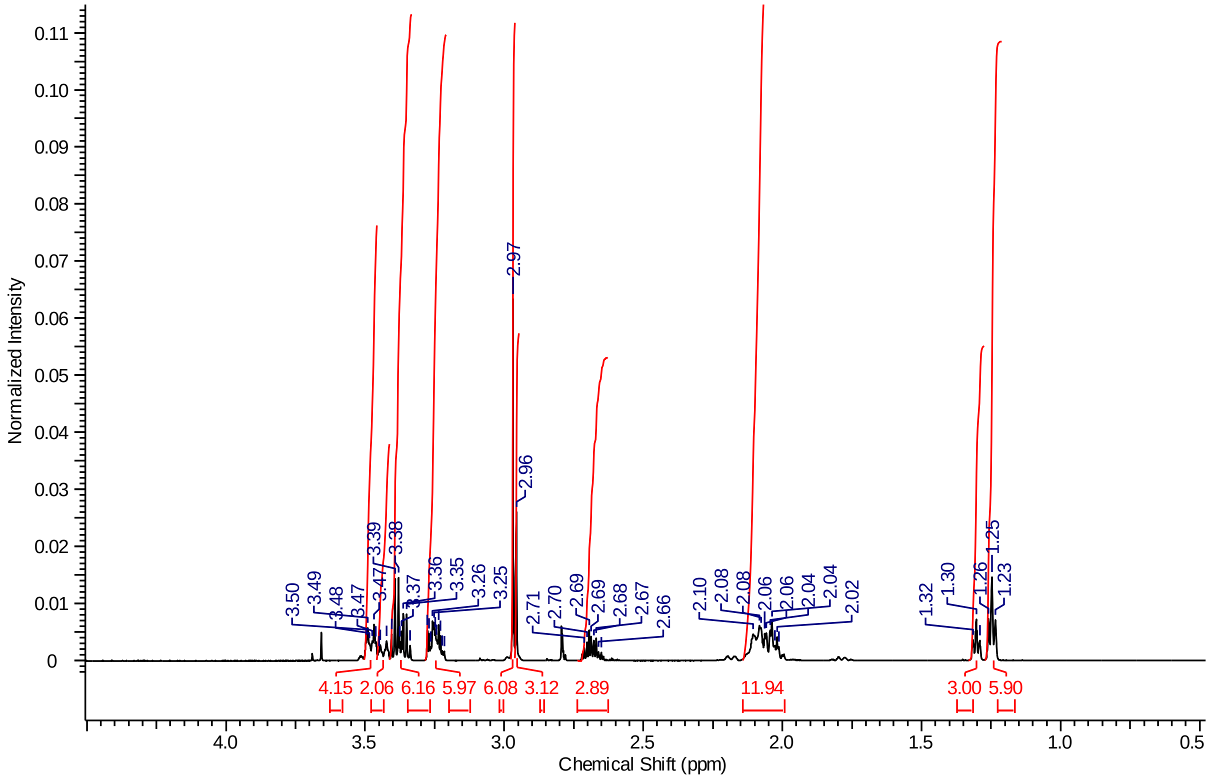

4-Carboxy-1,1-dimethyl-1,2,3,6-tetrahydropyridin-1-ium (7)

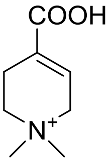

The trifluoroacetic acid (TFA) salt of desired product (14 mg, 0.05 mmol, 12%) was obtained starting from isonipecotic acid (50 mg, 0.39 mmol) by following the general procedure.

^{1}H NMR (700 MHz, D_2O) δ = 6.77 - 6.83 (m, 1 H), 4.07 (q, *J*=2.5 Hz, 2 H), 3.50 (t, *J*=6.5 Hz, 2 H), 3.10 (s, 6 H), 2.65 - 2.72 (m, 2 H) ppm, ^{13}C NMR (126 MHz, D_2O) δ = 168.4, 129.1, 127.6, 60.7, 58.2, 51.7, 19.5 ppm. HRMS (ESI-TOF) calcd for $C_8H_{14}NO_2^+$ [M^+]: 156.1019, found: 156.1013.

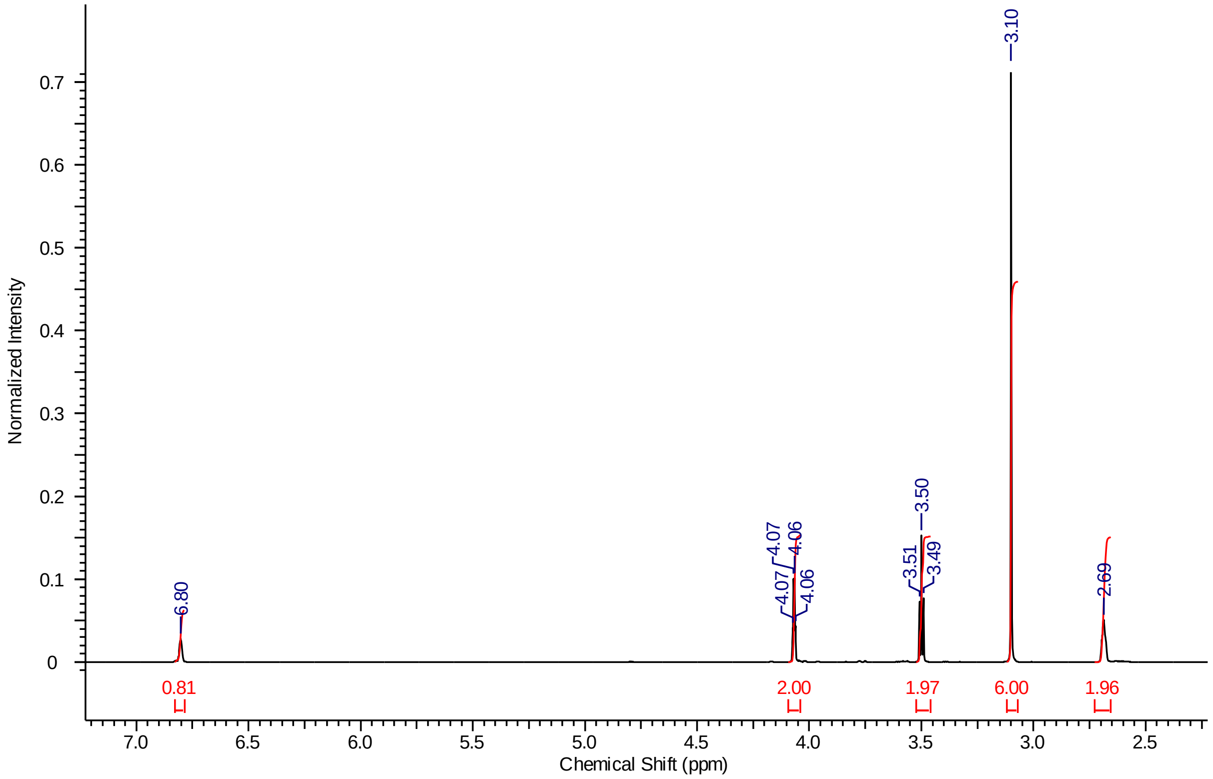

4-Carboxy-1,1,4-trimethylpiperidin-1-ium (8)

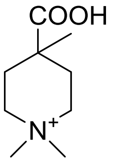

The trifluoroacetic acid (TFA) salt of desired product (10 mg, 0.03 mmol, 8%) was obtained starting from 4-methylisonipecotic acid (50 mg, 0.39 mmol) by following the general procedure.

^{1}H NMR (700 MHz, D_2O) δ = 3.47 (dt, *J*=13.5, 4.0 Hz, 2 H), 3.35 (td, *J*=3.0, 12.5 Hz, 2 H), 3.16 (s, 6 H), 2.32 (d, J=15.5, 2 H), 1.95 (ddt, J=3.5, 11.0, 15.5, 2 H), 1.35 (s, 3 H) ppm, ^{13}C NMR (126 MHz, D_2O, from HSQC signals) δ = 59.8, 54.3, 49.2, 28.2, 24.2 ppm. HRMS (ESI-TOF) calcd for $C_9H_{18}NO_2^+$ [M^+]: 172.1332, found: 172.1325.

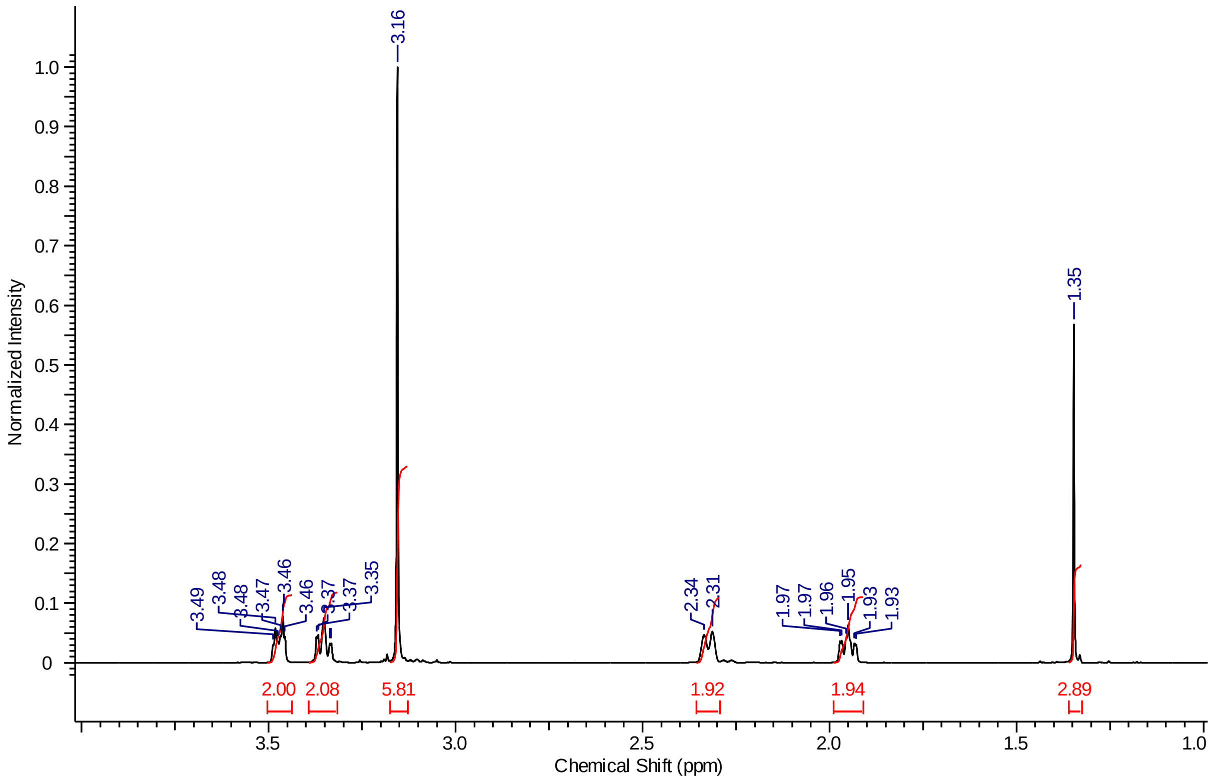

4-Carboxy-1-methylquinuclidin-1-ium (9)

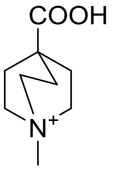

The trifluoroacetic acid (TFA) salt of desired product (16 mg, 0.05 mmol, 13%) was obtained starting from 1-aza-bicyclo[2.2.2]octane-4-carboxylic acid (50 mg, 0.39 mmol) by following the general procedure.

^{1}H NMR (700 MHz, D_2O) δ = 3.47 (t, *J*=7.5 Hz, 6 H), 2.96 (s, 3 H), 2.17 (t, *J*=7.5 Hz, 6 H) ppm, ^{13}C NMR (126 MHz, D_2O, from HSQC signals) δ = 56.3, 51.6, 25.8 ppm. HRMS (ESI-TOF) calcd for $C_9H_{18}NO_2^+$ [M^+]: 170.1176, found: 170.1179.

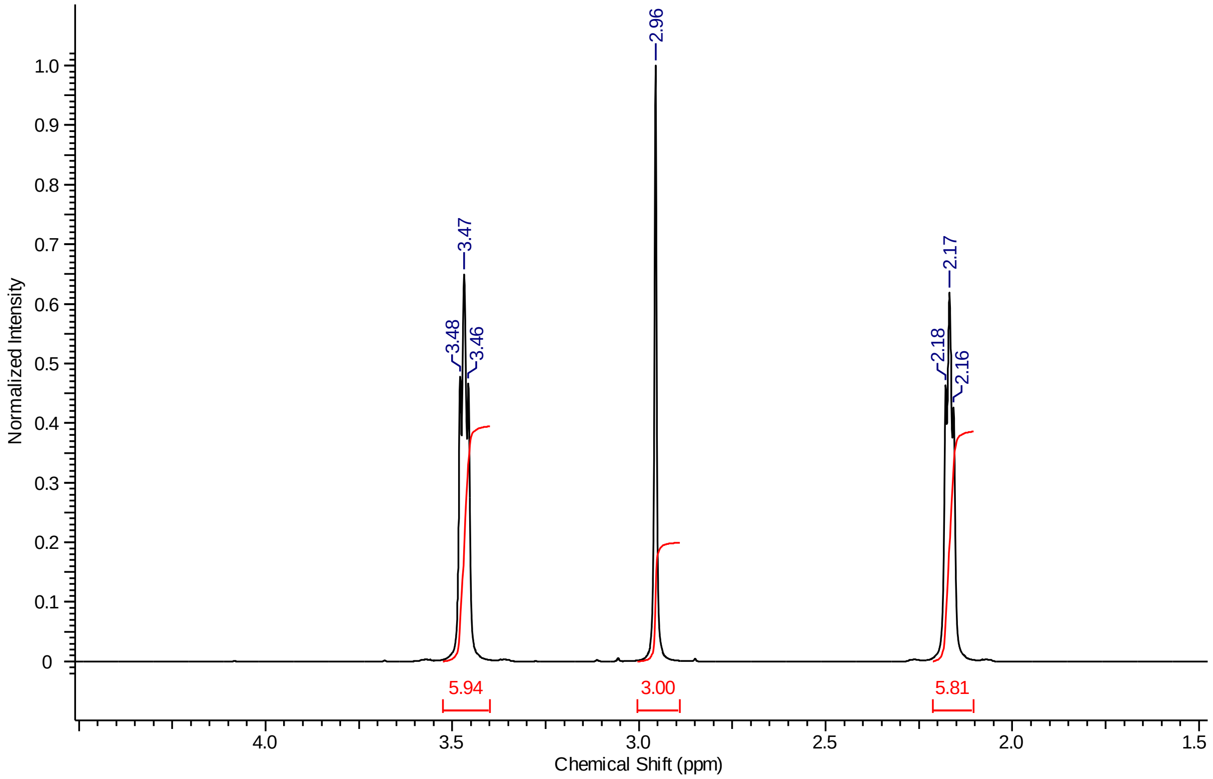

(*S*)-3-Carboxy-1,1-dimethylpyrrolidin-1-ium (10)

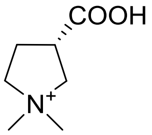

The trifluoroacetic acid (TFA) salt of desired product (10 mg, 0.04 mmol, 9%) was obtained starting from (*S*)-pyrrolidine-3-carboxylic acid (50 mg, 0.43 mmol) by following the general procedure.
^{1}H NMR (700 MHz, D_2O) δ = 3.72 - 3.82 (m, 2 H), 3.52 - 3.62 (m, 3 H), 3.15 (s, 3 H), 3.12 (s, 3 H), 2.53 - 2.62 (m, 1 H), 2.39 - 2.47 (m, 1 H) ppm, ^{13}C NMR (126 MHz, D_2O) δ = 175.5, 66.4, 65.3, 40.5, 52.2, 26.5 ppm. HRMS (ESI-TOF) calcd for $C_7H_{14}NO_2^+$ [M^+]: 144.1019, found: 144.1013.

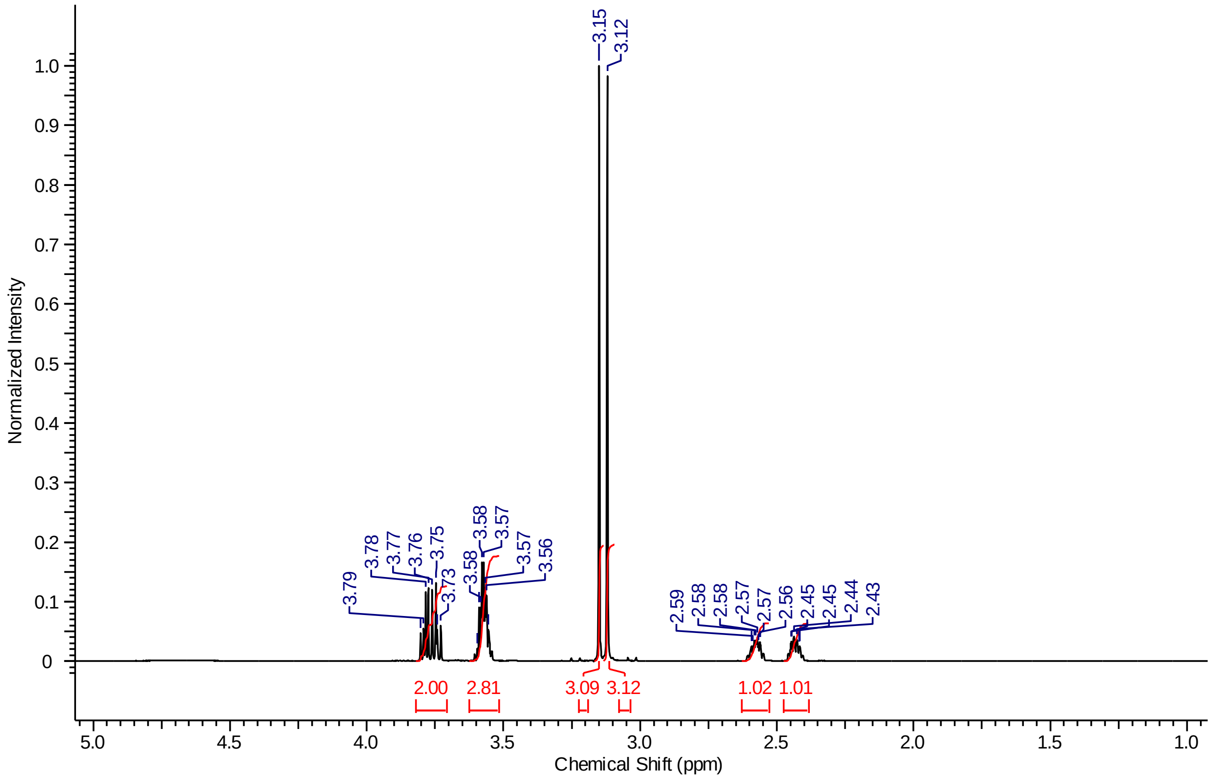

(R)-3-Carboxy-1,1-dimethylpyrrolidin-1-ium (11)

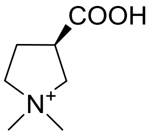

The trifluoroacetic acid (TFA) salt of desired product (13 mg, 0.05 mmol, 12%) was obtained starting from (*R*)-pyrrolidine-3-carboxylic acid (50 mg, 0.43 mmol) by following the general procedure. ^{1}H NMR (700 MHz, D_2O) δ = 3.72 - 3.82 (m, 2 H), 3.52 - 3.62 (m, 3 H), 3.15 (s, 3 H), 3.12 (s, 3 H), 2.53 - 2.62 (m, 1 H), 2.39 - 2.47 (m, 1 H) ppm, ^{13}C NMR (126 MHz, D_2O) δ = 175.5, 66.4, 65.3, 40.5, 52.2, 26.5 ppm. HRMS (ESI-TOF) calcd for $C_7H_{14}NO_2^+$ [M$^+$]: 144.1019, found: 144.1019.

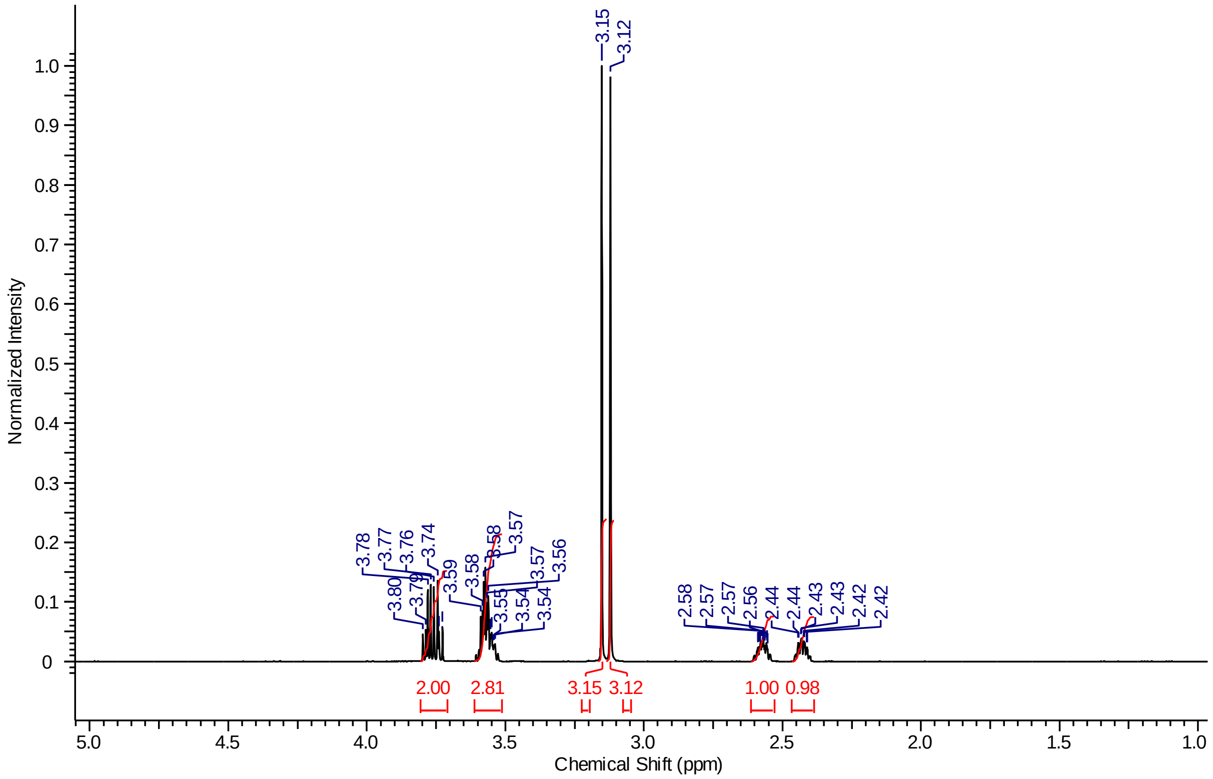

(R)-2-Carboxy-1,1-dimethylpyrrolidin-1-ium (12)

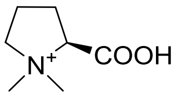

The trifluoroacetic acid (TFA) salt of desired product (10 mg, 0.04 mmol, 9%) was obtained starting from (*R*)-proline (50 mg, 0.43 mmol) by following the general procedure.

^{1}H NMR (700 MHz, D_2O) δ = 4.27 (t, *J*=9.5 Hz, 1 H), 3.68 (ddd, *J*=11.5, 8.5, 3.5 Hz, 1 H), 3.53 (q, *J*=9.5 Hz, 1 H), 3.27 (s, 3 H), 3.07 (s, 3 H), 2.46 - 2.54 (m, 1 H), 2.28 - 2.36 (m, 1 H), 2.07 - 2.20 (m, 2 H) ppm, ^{13}C NMR (126 MHz, D_2O) δ = 169.0, 74.2, 67.8, 52.2, 46.2, 24.6, 18.5 ppm. HRMS (ESI-TOF) calcd for $C_7H_{14}NO_2^+$ [M^+]: 144.1019, found: 144.1015.

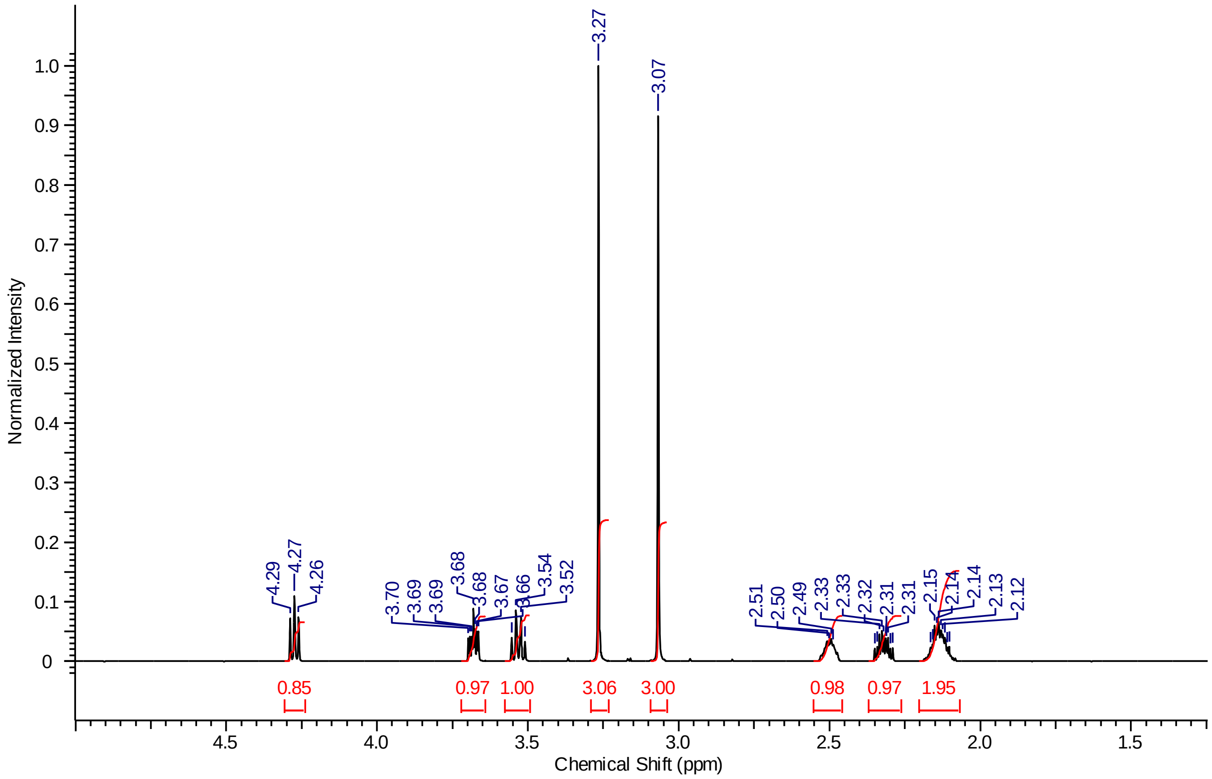

3-(Carboxymethyl)-1,1-dimethylpiperidin-1-ium (13)

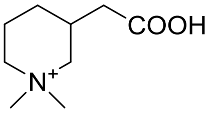

The trifluoroacetic acid (TFA) salt of desired product (10 mg, 0.03 mmol, 9%) was obtained starting from (*S*)- 3-piperidinecarboxylic acid (50 mg, 0.35 mmol) by following the general procedure.

^{1}H NMR (700 MHz, D_2O) δ = 3.41 (t, *J*=15.5 Hz, 2 H), 3.15 (t, *J*=13.0 Hz, 1 H), 3.08 (s, 3 H), 3.05 (s, 3 H), 2.94 (t, *J*=12.5 Hz, 1 H), 2.36 - 2.44 (m, 1 H), 2.31 (qd, *J*=16.5, 6.5 Hz, 2 H), 1.87 - 1.98 (m, 2 H), 1.79 - 1.87 (m, 1 H), 1.16 (qd, *J*=13.0, 4.0 Hz, 1 H) ppm, ^{13}C NMR (126 MHz, D_2O) δ = 175.6, 65.8, 62.4, 56.7, 47.4, 37.2, 27.9, 26.8, 19.7 ppm. HRMS (ESI-TOF) calcd for $C_9H_{18}NO_2^+$ [M$^+$]: 172.1332, found: 172.1332.

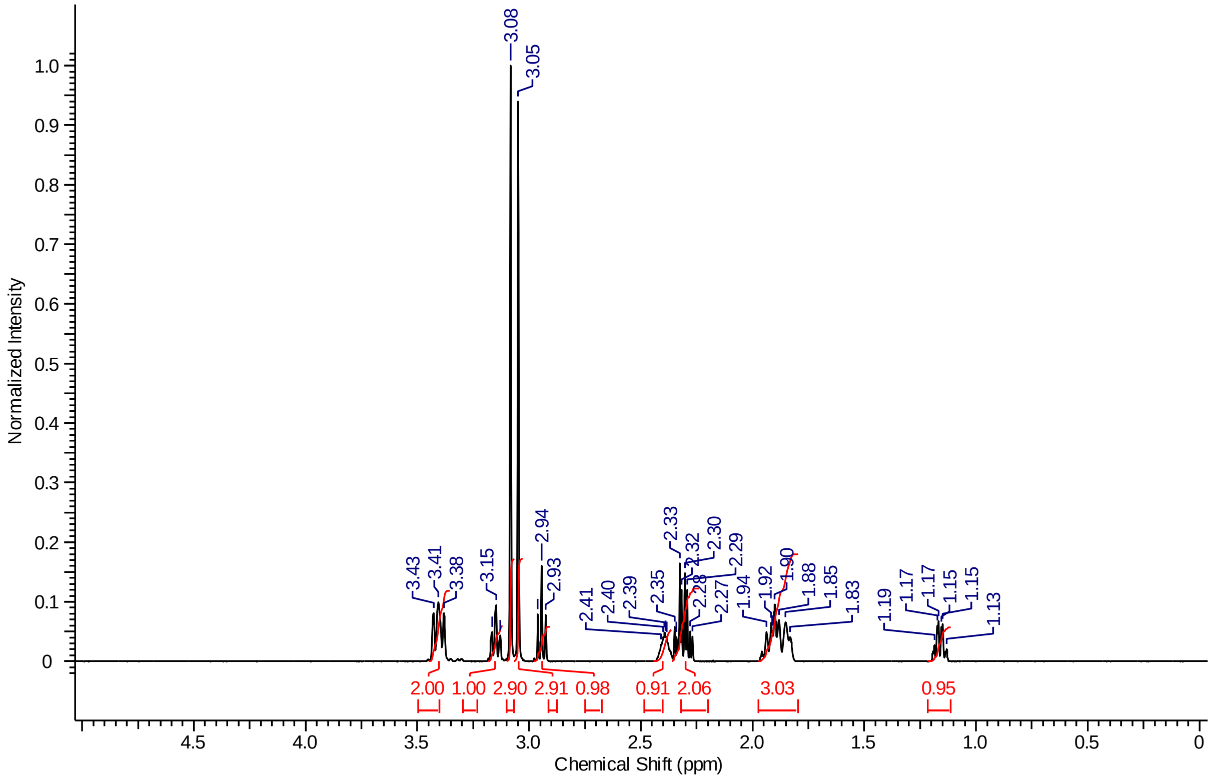

4-Carboxy-*N*,*N*,*N*-trimethylcyclohexan-1-aminium (14)

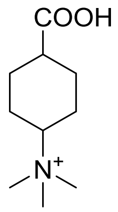

The trifluoroacetic acid (TFA) salt of desired product (11 mg, 0.04 mmol, 11%) was obtained starting from isonipecotic acid (50 mg, 0.35 mmol) by following the general procedure.

^{1}H NMR (700 MHz, D_2O) δ = 3.28 (tt, *J*=11.5, 3.5 Hz, 1 H), 2.97 (s, 9 H), 2.71 (td, *J*=4.5, 2.5 Hz, 1 H), 2.25 (d, *J*=14.0 Hz, 2 H), 2.06 - 2.12 (m, 2 H), 1.52 - 1.64 (m, 4 H) ppm, ^{13}C NMR (126 MHz, D_2O) δ = 178.8, 73.7, 50.5, 36.8, 25.4, 22.3 ppm. HRMS (ESI-TOF) calcd for $C_{10}H_{20}NO_2^+$ [M^+]: 186.1489, found: 186.1482.

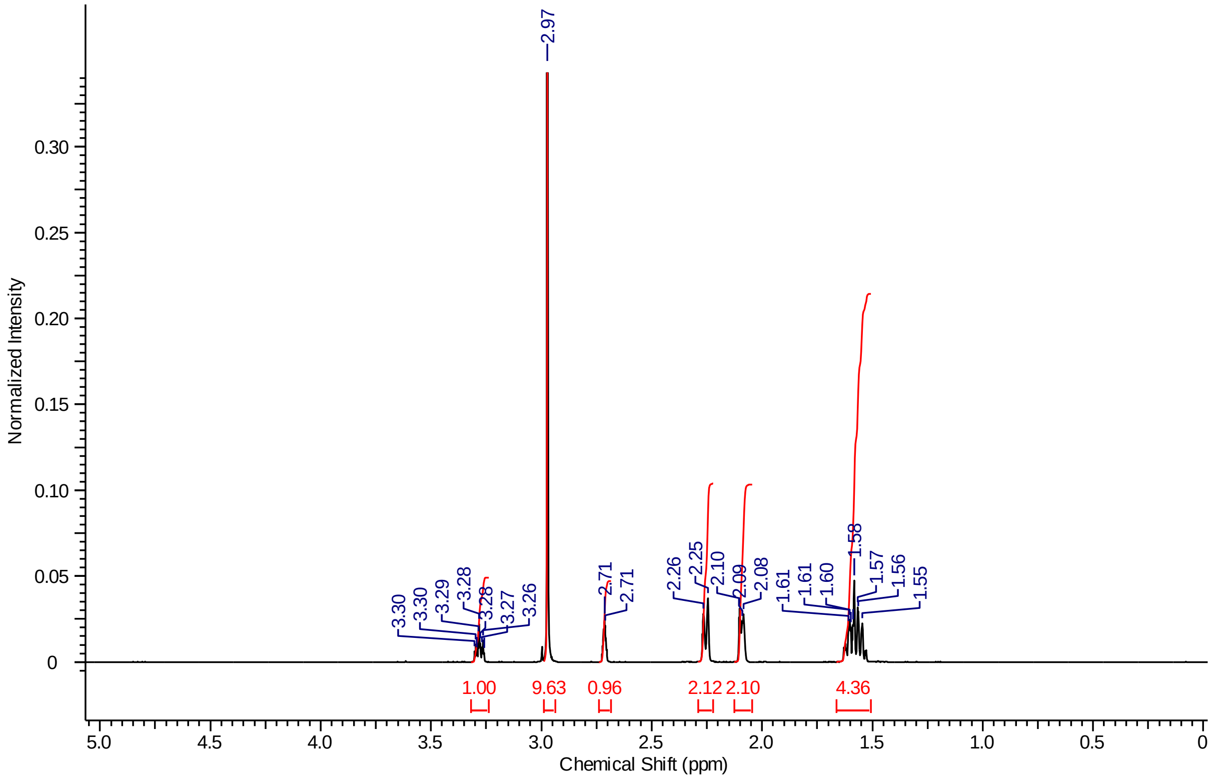

References

[1] I. K. H. Leung, T. J. Krojer, G. T. Kochan, L. Henry, F. von Delft, T. D. W. Claridge, U. Oppermann, M. A. McDonough, C. J. Schofield, *Chem. Biol.* **2010**, *17*, 1316-1324.

[2] A. M. Rydzik, I. K. H. Leung, A. Thalhammer, G. T. Kochan, T. D. W. Claridge, C. J. Schofield, *Chem. Comm.* **2014**, *50*, 1175-1177.

[3] Z. Otwinowski, W. Minor, C. W. C. Jr., in *Methods in Enzymology, Vol.* ***276***,, Academic Press, **1997**, pp. 307-326.

[4] A. J. McCoy, R. W. Grosse-Kunstleve, P. D. Adams, M. D. Winn, L. C. Storoni, R. J. Read, *J. Appl. Crystallogr.* **2007**, *40*, 658-674.

[5] P. Emsley, K. Cowtan, *Acta Crystallogr. D Biol. Crystallogr.* **2004**, *60*, 2126-2132.

[6] P. D. Adams, P. V. Afonine, G. Bunkoczi, V. B. Chen, I. W. Davis, N. Echols, J. J. Headd, L.-W. Hung, G. J. Kapral, R. W. Grosse-Kunstleve, A. J. McCoy, N. W. Moriarty, R. Oeffner, R. J. Read, D. C. Richardson, J. S. Richardson, T. C. Terwilliger, P. H. Zwart, *Acta Crystallogr. Sect. D* **2010**, *66*, 213-221.
